# Industry-University Collaborations in Canada, Japan, the UK and USA – With Emphasis on Publication Freedom and Managing the Intellectual Property Lock-Up Problem

**DOI:** 10.1371/journal.pone.0090302

**Published:** 2014-03-14

**Authors:** Robert Kneller, Marcel Mongeon, Jeff Cope, Cathy Garner, Philip Ternouth

**Affiliations:** 1 University of Tokyo, Research Center for Advanced Science and Technology (RCAST), Tokyo, Japan; 2 Ross, Mongeon, Covello & Co., London, Ontario, Canada; 3 Office of Research Contracts and Intellectual Property, McMaster University, Hamilton, Ontario, Canada; 4 Research Triangle Institute, Research Triangle Park, North Carolina, United States of America; 5 Manchester Knowledge Capital, Ltd., Manchester, United Kingdom; 6 Council for Industry and Higher Education, London, United Kingdom; 7 Council for Industry and Higher Education, London, United Kingdom; Universidad Veracruzana, Mexico

## Abstract

As industry-university collaborations are promoted to commercialize university research and foster economic growth, it is important to understand how companies benefit from these collaborations, and to ensure that resulting academic discoveries are developed for the benefit of all stakeholders: companies, universities and public. Lock up of inventions, and censoring of academic publications, should be avoided if feasible. This case-study analysis of interviews with 90 companies in Canada, Japan, the UK and USA assesses the scope of this challenge and suggests possible resolutions. The participating companies were asked to describe an important interaction with universities, and most described collaborative research. The most frequently cited tensions concerned intellectual property management and publication freedom. IP disagreements were most frequent in the context of narrowly-focused collaborations with American universities. However, in the case of exploratory research, companies accepted the IP management practices of US universities. It might make sense to let companies have an automatic exclusive license to IP from narrowly defined collaborations, but to encourage universities to manage inventions from exploratory collaborations to ensure development incentives. Although Canada, the UK and US have strong publication freedom guarantees, tensions over this issue arose frequently in focused collaborations, though were rare in exploratory collaborations. The UK Lambert Agreements give sponsors the option to control publications in return for paying the full economic cost of a project. This may offer a model for the other three countries. Uniquely among the four countries, Japan enables companies to control exclusively most collaborative inventions and to censor academic publications. Despite this high degree of control, the interviews suggest many companies do not develop university discoveries to their full potential. The steps suggested above may rebalance the situation in Japan. Overall, the interviews reveal the complexity of these issues and the need for flexibility on the part of universities and companies.

## Introduction

Links between universities and industry are important mechanism to develop and commercialize the fruits of university research. Such links are also seen as contributing to technological progress and economic well being [1], [2]. Most studies have examined these linkages from the perspective of university researchers and administrators [3]. This report examines these linkages from the perspective of industry, focusing particularly on issues related to intellectual property (IP) arising from collaborations with universities, and upon the freedom of academic researchers to publish findings arising from these collaborations. It also compares university policies governing these issues in the four countries that are the subject of this survey, Canada, Japan, the UK and the USA. It examines how tensions vary according to the the type of collaboration (narrowly/focused vs. broad/exploratory), the type of company (large vs startup or other SME), and the legal and institutional environment.

The analysis is based upon interviews with 90 companies conducted in 2008. It is an exploratory analysis. However, it does suggest possible approaches to managing IP and publication freedom issues that might satisfy the core interests of stakeholders in various situations.

The initial analysis of these case studies, completed in 2009, found that, among the various types of industry-university linkages, collaborative research is the most important for developing university discoveries and contributing to economic growth. It also found that policies governing rights to IP arising under collaborative research, and the rights of university researchers to publish findings from such research, are often important considerations for both parties and are the sources of most major disputes between the collaborative research parties.

The framework for this paper is a comparative analysis — based upon this relatively large number of case studies — of these policies in each of the four countries and how their impact varies according to the type of research and types of companies involved. While the policy suggestions it offers are only tentative, they draw upon the insights gained from the unique nature of this study.

## Background and Related Studies

### 1. Overview of Industry Motivations to Collaborate with Universities

The original study was conceived against the backdrop of the rich literature about engagement of university researchers with industry and the commercialization of university discoveries. Although most of these studies have been from the perspective of universities, some did examine industry perspectives [3]. This subset of studies pointed out that successful collaboration outcomes often depend upon trust and communication [4], [5], as well as corporate management's commitment to the collaboration [6]. Motivations for companies to engage with universities include accessing complementary research expertise for future business development [7]–[9], particularly for products that are in the design or early development stage [10]. In contrast to large companies, small or new companies tend to rely on universities for their core technologies [8], [11].

### 2. Findings from the Initial Analysis of Interview Responses

This study was originally undertaken to deepen understanding from these previous studies, particularly with respect to what types of engagements are most beneficial for industry, the process of embedding knowledge from universities, and the challenges encountered in managing relationships with universities. Data were collected by structured interviews in 2008 with 90 companies in Canada, Japan, the UK, and USA. A list of the companies covered by the interviews and the collaborating universities appears in Table S1.

An initial analysis of the findings was presented in March 2009 at the University of California, San Diego (UCSD) in the workshop, “What Industry Wants from Universities” sponsored by the Kauffman Foundation, the Sasakawa Peace Foundation, and the Council for Industry and Higher Education (CIHE) in the UK [12].

The most important finding in the original report is that the vast majority (90 percent) of the interactions were collaborative research projects involving company and university researchers, not simple license agreements. Among all 90 interviews, only two cited interactions that were limited mainly to licensing. This finding is consistent with industry funding for sponsored university research being considerably higher than university licensing revenue: $4.1 billion vs. $2.6 billion, respectively, in 2012 for American universities, with the proportionate imbalance in favor of sponsored research even greater in the other three countries [Note S1].

The 2009 report also emphasized the importance of individuals in both universities and companies who can communicate needs and capabilities between organizations and within organizations. It stressed their roles as boundary spanners between organizations and in increasing the absorptive capacity of companies.

Theses findings coincide with those by other researchers. For example, a recent interview survey of industry managers engaged in Faraday Partnerships with UK universities found that actual research engagement and boundary spanners were more important than licensing [7]. Mid-range universities in Belgium and the UK contribute most to innovation and regional economic development via production of skilled graduates, start-ups, and contract research, not licenses [17]. A 1994 survey of US industry R&D managers found that “In most industries, patents and licenses are not nearly as important as other channels for conveying public research to industry.” It concluded that face-to-face interactions, such as under joint or cooperative ventures, complement the more public channels of information transfer, such as publications and meetings, that are the most important channels for transferring university knowledge to industry [10]. Our finding that industry values collaborative research most among all forms of university interactions is consistent with reports of increasing collaborations involving pharmaceutical companies [18], the experience of particular institutions such as Pennsylvania State University [19]–[20], and recent increases in industry sponsorship of university research in all four countries [13]–[16], [21].

The 2009 report also described how successful collaborations often grew out of a previous interaction or personal connections, such as those provided by faculty founders of startups, professional networks, or professor-student relationships. However, previous connections do not always guarantee success and some collaborations are initiated de novo [Note S2].

Another conclusion of the 2009 analysis was that champions within universities and especially companies are vital to the success of industry-university collaborations and their long-term continuation. This was supported by interviews with industry R&D managers engaged in collaborations with Pennsylvania State University [20] as well as studies in Spain [22] Sweden and Australia [6].

### 3. Re-examination of Interviews and Focus on IP and Publication Freedom

Beginning in late 2011, an analysis of the transcripts of each of the 90 interviews was undertaken to look for additional insights pertaining to how industries benefit from relationships with universities, impediments to these relationships, and how these impediments might be resolved. It became apparent that the main barriers involved friction with the universities over issues relating to IP rights and academic publication freedom. Thus, these two issues became the focus of this deeper examination of the interviews, with the basic framework of analysis being national and university policies related to IP management and publication freedom, in the context of collaborative research.

#### 3.1 Previous studies focusing on IP issues

Previous studies have also indicated that IP issues and institutional bureaucracy are often stumbling blocks in industry-university collaborations. An early survey of science laboratories in Belgian universities found that individual initiative by professors and industry counterparts is the most important factor in starting collaborations, and that formal institutional initiatives (particularly liaison offices with responsibilities ranging from initiating collaborations to IP management to startup creation) often get in the way. It stressed the effectiveness of direct communication and meeting of minds between industry and university researchers, and attributed the ineffectiveness of formal institutional initiatives to poorly defined goals and lack of staff competence [23]. Interviews with 59 English startups and other SMEs in the 1990s suggested that universities' emphasis on IP ownership and management was misplaced, because small English firms simply were not able to enforce IP rights and thus do not attach much value to them. Open communication and trust with university researchers was more important, but as universities began to formalize IP management this threatened to interfere with these informal, trust-based relationships [24]. A survey of the lead corporate participants in 38 projects funded by the US Advanced Technology Program (ATP) between 1993 and 1996 found that one third of these companies conscientiously decided not to include a university in their project because of “insurmountable IP obstacles”. The companies often cited university resistance to the companies' expectation that they should own resulting IP, or should at least have exclusive rights to such IP. However, this survey also found that companies with more experience working with universities were less likely to cite insurmountable obstacles [2]. Hertzfeld et al [25] obtained survey responses from 54 mainly large American firms that had entered into research joint ventures from 1995 through 1998. The respondents acknowledged that patent rights were generally an important consideration, and that IP negotiations are sometimes complex, although they rarely present insurmountable obstacles. However, they felt that IP issues were most problematic when dealing with universities, and there was a trend for universities to be “more aggressive” and “greedy” when negotiating issues related to joint venture IP. Lack of understanding of business, overvaluation of their inventions, inexperience, and taking too long to make a deal, were criticisms frequently leveled at university IP management offices.

However, at least one study has concluded that universities generally do not extract as much economic benefit from their biotechnology licensing agreements as they could [26]. Another line of research indicates that universities that allocate a greater proportion of license revenues to their faculty/student inventors have higher total license revenues, suggesting that academic researchers are motivated by monetary rewards to make commercially valuable inventions [27].

In response to concerns that negotiations over intellectual property arising from collaborations between universities and IT companies were taking too long to negotiate and were interfering with collaborations, the Kauffman Foundation and several American IT companies and universities put forward Open Innovation Principles in 2005. These state that IP covering software inventions arising from industry-university collaborations should be made freely available to anyone without need for a license [Note S3].

#### 3.2 Previous studies focusing on publication freedom

Concerns over publication freedom were illustrated by events such as Boots Pharmaceutical's attempt to prevent publication of clinical research from the University of California at San Francisco showing that its brand name formulation of levothyroxine, Synthroid, was no more effective than generic versions of the drug for thyroid hormone replacement therapy [28]. Following a similar case involving a University of Toronto researcher, that University and all its affiliated hospitals announced a policy to prohibit clauses in sponsored research contracts that allow the sponsors to “suppress or otherwise censor research results.” [29], [30] (For additional cases, see [31], [32].) Around the same time, a survey of over 2000 academic life scientists found that twenty percent had delayed publications by over six months, 28 percent of these in order to “slow dissemination of undesired results” [33]. The authors of this study intended this category to cover instances of sponsor-imposed delays, such as the Synthroid case, as well as self-censorship [34]. As discussed below, the majority of American and Canadian research universities now have written policies that permit publication delays for only limited periods (usually less than six months) and only to provide enough time to ensure that none of the sponsor's own confidential information is revealed and to prepare patent applications. However, a follow up survey of US life science faculty in 2006 found that five percent of those receiving industry funding reported having a publication delayed more than six months in order to “inhibit the dissemination of undesired results.” In contrast, only one percent of those not receiving industry funding experienced such delays. The authors of this study concluded “data withholding remains a greater (although perhaps diminishing problem) for industry funded scientists.” [35]

One of the few studies to examine the perspective of companies towards American university publication policies suggested that relatively few problems arose during negotiations. “A compromise on this issue (usually in the form of a delay in publication until IP rights are secured) seems to be acceptable to researchers, universities and sponsoring companies [25].” On the other hand, companies with research ties to Pennsylvania State University stated in interviews that disagreements related to IP and publication freedom are two of the main barriers to collaborations [19], [20]. In addition, the Canadian Association of University Teachers (CAUT) recently criticized Canadian universities for not including specific protections for academic freedom in a majority of the industry collaborative agreements they reviewed. CAUT Principles issued in 2012 call for explicit protections of academic freedom in every donor/collaboration agreement and a strict 60-day limit on any publication delays. Furthermore, they declare, “Any interference with a researcher's right and responsibility to publish results, regardless of effect on the collaborating organization, is unacceptable.” A professor leading one of the collaborative projects responded that academics who voluntarily take industry funding ought to have some accountability to the companies that support their research. [36], [37]

As discussed below, UK universities have addressed industry control over academic publications through the Lambert Model Agreements. In Korea, university researchers and companies apparently agree that industry sponsors ought to be able to require deletions from academic publications of sponsored research findings [38].

Against the background of this previous scholarship and the ongoing debate about the scopes of publication freedom and sponsors' control over IP in the context of collaborative research, our study revisits these issues – with the objective of suggesting ways university policies can adjust to different situations to try to satisfy the core interests of all parties involved.

## Methods

As an exploratory study, the method selected to address the above issues was to interview a cross section of companies in each of four countries and to request that the interviewees describe in depth an important interaction each had recently had with a university. These descriptions would provide insights into the sorts of interactions companies consider important, how they engage universities, the process of embedding and using university knowledge, the impact on the companies' value chains, and the how the environment for future collaborations can be improved. A semi-structured questionnaire was developed by the Council for Industry and Higher Education (CIHE) in the UK and the Centre for Business Research (CBR) of the Judge School of Business of Cambridge University and consisted of the following questions.

1. How was the requirement to interact with a university identified? If the opportunity resulted from initial contact with a university, how did this occur (e.g., web search, existing contact, contact from university, networking events, conference)? What alternatives were considered?
2. What were the origins of the knowledge transferred and how would it/they be classified? Was this part of a sequence of activities with the university or a single self-contained project?
3. What internal justification in the company was needed to work with the university?
4. What transactions need to occur to support and give legal effect to the project knowledge transfer (e.g. IP licensing or non-disclosure agreements (NDAs))?
5. How did the negotiations to set up the project proceed? How were the negotiations managed and, where there was a financial component (e.g. a research sponsorship, license agreement), what was your perception of how the university approached this? What were the perceptions of value on each side and how were they assessed? Were there any issues arising in the negotiations and how were they resolved?
6. How did the project proceed? i.e. What was the nature of the interaction (e.g. academic working in the company, work done in university with results transferred via report)? By what mechanism did the knowledge transferred become embedded in the company? What was the nature of the embedding (e.g. certain key individuals with new skills and knowledge)? How did the company know that this had occurred? What did the company need to do subsequently to realize the potential value generated by the project?
7. What was the nature of the impact upon the company value chain (i.e. direct contribution of technology to product development, manufacturing or logistics process, upskilling/increasing knowledge of staff, service development)? How easy was it to identify?
8. What was the nature and scale of the outcome (e.g. increased sales, new markets, faster to market, more efficient process) and how was it evaluated?
9. Has this project affected the company's potential to collaborate with universities?

These questions were developed by drawing upon earlier studies by CIHE and CBR to assess the impact on regional competitiveness of higher education institutions [39], [40]. These earlier studies represent an initial scoping phase of this study funded by the UK Economic and Social Research Council (ESRC). The ESRC also funded the UK portion of this main study, while the Sasakawa Peace Foundation funded the interviews and analysis in the Canada, Japan and the USA.

It was decided that the respondents would be senior officials responsible for research management for the entire corporation or for the main corporate laboratory that interacted most frequently with universities (usually the basic or central research laboratory). Respondents could also be persons responsible for corporate business development, if their remit covered interactions with universities. In other words, respondents were persons who had a corporate wide perspective, and who were familiar with the technical aspects of their company's operations and the extent to which their companies engaged in technical collaborations with universities. In the case of some companies, the unit of analysis was the operations in a particular country of a large corporation headquartered in another country.

The following partners, selected for their familiarity with the prevailing legislative, business and academic environments in each of their countries, were responsible for selecting the companies, identifying respondents, conducting the interviews and country-specific analysis for each of their respective countries:

- For the UK – CIHE,
- For the USA, RTI International,
- For Canada, Mongeon Consulting,
- For Japan, the Research Center for Advanced Science and Technology (RCAST) in the University of Tokyo (Principal Investigator, Professor Robert Kneller).

Partners had flexibility in devising selection methods, while adhering to the guideline that the selected companies approximate a representative cross section of companies engaged in significant interactions with universities. In the UK, the CIHE used its list of members as a base from which to select companies. In addition, the CIHE was able to use some of its previous interviews conducted in collaboration with the CBR to add to the pool of case studies. The Canadian partner, Mongeon Consulting, had a list of possible interview targets through past experience providing IP advice and technology transfer services to Canadian universities and companies. The US partner, RTI International, is a leading independent, nonprofit research and development organization. RTI was formed in 1958 by the three principal universities in Research Triangle Park: University of North Carolina, North Carolina State University and Duke University. Since then it has undertaken research projects on behalf of many other universities and companies, and from these it developed its sampling frame. The Japanese partner, Robert Kneller in the University of Tokyo, had previously done case study interviews of Japanese startups and pharmaceutical companies, and had been compiling news reports on industry-university collaborations from the Nikkei Financial Daily and similar sources. His basic sample frame was the list of companies appearing in such reports dating from January 2007. He engaged the non-profit research organization, “Institute for Future Technology (IFTECH)” to help draft the questionnaire and introductory materials in Japanese. IFTECH also contacted companies to request and arrange interviews. Almost all the interviews involved Prof. Kneller and a member of IFTECH, and almost all were in Japanese.

In all four countries, interviews were granted by the majority of companies contacted by the partners that acknowledged important interactions with universities. Not surprisingly, in view of the criterion that these interactions have been important for the company, most of projects described were deemed successful. While this means that the case studies are less likely to be representative of industry-university interactions as a whole (because failures tended not to be highlighted) they help to illustrate the actual path to market of university knowledge. In some cases, a company identified several projects or a family of projects that arose from a common mode of interaction with universities.

Altogether the teams conducted 90 interviews, 20 with Canadian companies, 20 with US companies, 21 with Japanese companies and 29 with UK companies. However, some of these interviews highlighted another company. For example, the interview with an electronics company concerning its collaboration with Cambridge University's Cavendish Laboratory featured a spin-off from this collaboration, TeraView. Thus TeraView is also listed as one of the UK interviewee companies. Such companies are designated by “†” in Table S1. Sometimes a company described a single initiative involving two or more universities. For example, General Mills began a collaboration with MIT to expand production efficiency and then expanded this to include the University of Minnesota. Three of the Japanese respondents all happened to describe a collaboration with a single Japanese university with which they were all collaborating in the same consortium project. Relatively frequently, a company described more than one collaborative project, each involving different universities. Other companies described a general collaborative initiative and did not give examples of projects with specific universities. In such cases the academic partners were listed as “various” or “unidentified,” and if some universities were mentioned only in passing, such universities were identified with “*”. Several universities were identified as significant collaborators in several interviews, each dealing with an unrelated project.

Within a few days of each interview, a detailed report was compiled. For this paper, one of the authors (RK) read through each report and summarized key points from each interview, such as the nature of the collaboration, how it was initiated, the contribution of the company, the contribution of the university, intellectual property and secrecy issues, how knowledge was embedded, and benefits for the company. For each of the latter three categories, an indicator of positiveness was assigned on a five-point scale with 5 being most positive. In addition, RK attempted to find out independent information about the collaboration. Very often this additional information helped to clarify the interaction, put it in context with the company's overall business strategy, and update information about the interaction. In a few cases, follow-up interviews were conducted.

In the remainder of this paper, the main text is devoted to analyzing the case reports, while summaries of relevant case studies are presented in Supporting Information files designated as Case S1, Case S2, etc. Cases were highlighted in this manner if they illustrated an interesting phenomenon or were representative of a recurring theme, provided that highlighting would neither embarrass the company or university, nor reveal sensitive, non-public information.

The following caveats should be kept in mind. First, although the total number of cases is relatively large, this is far from a comprehensive survey and any discussion of comparative rates between countries or industries, is speculative. Simply put, we lack appropriate denominators for much of the analysis. Second, as already mentioned, the interviews generally described successful collaborations and thus are not representative of the entire universe of interactions. Nevertheless, they do provide a wide cross-section of positive and negative viewpoints on IP and confidentiality issues. Third, the interview data are now somewhat dated. However, because the second-look analysis involved updating the current status of the highlighted activities (to the extent information is available) we have a fair idea of whether some of the projects have been closed down or have borne fruit. Finally, many of the interviews were granted only on condition that certain information not be publicly disclosed. In several cases, a company did not disclose the identity of the collaborating university or the project. In one case a company did not want even its identity disclosed to other study team members. Thus companies and universities are generally not identified by name. All the companies that were the subject of interviews are listed in Table S1 as are all the collaborating universities. However these lists are alphabetical with information about pairings removed.

## Classification and Distribution of the Interactions

As shown in Table 1, 80 of the 90 interactions (90 percent) involved some sort of collaborative or commissioned research, i.e., projects where the company sought new research conducted by university personnel. In fact, they were probably all collaborative (a.k.a., Joint research) projects, because they all involved interaction between company personnel and the university researchers, with the possible exception of the last project listed under *atypical cases* below. In other words, none of these 80 cases centered on a company simply contracting with a university to prepare a written report. However, they did include the following atypical cases:

**Table 1:** All 90 cases classified by country and type of interaction.

	Canada	Japan	UK	USA	Total
Collaborative (or commissioned) research (includes atypical cases)	18	18	27	17	80
Training to meet needs of company's industry	1			1	2
A license was the main aspect of the interaction	1			1	2
Engaging universities as clients for company's business		3	1		4
Recruitment only			1		1
Business development, technology commercialization				1	1
Total	20	21	29	20	90

- Two cases without contractual agreements with the universities where the researchers were officially engaged as consultants. One involved development of a line of sophisticated scientific instruments that allowed a UK company to adopt these as a new line of business [Case S1]. The other involved a large electronics company engaged in a range of projects with US universities that are officially structured as consultancies so to avoid university claims to resulting IP.
- An endowed chair in a Canadian university for research & training related to automated inspection of food during processing. [Case S2]
- An interaction that began with a company trying to sell its software to a UK university but ended up with the company becoming involved in applications-oriented research and a university spin-off. [Case S3]
- An interaction that began with a company consulting for a US university to improve the university's manufacturing technology programs, but which evolved into a relationship where the company could increase its awareness of cutting edge manufacturing issues and better target its collaborative research projects with this and other universities.
- An interaction with a UK grocery retailer focused on supply chain management issues. The main participants were the chain's suppliers, and the interaction involved training courses as well as research. [Case S4]
- Identifying an academic consultant and host for a radio broadcast series
- A UK insurance company helping to establish a network of academics, government and industry persons to encourage more academic analysis of risks relevant to the insurance industry.

Even subtracting these eight atypical cases, collaborative research would still constitute 80% of the cases.

The next largest category was interactions aimed at making the university a business client (Table 1). One variation on this was the company engaging universities in order to become the marketing agent for some of their technologies. Most of these involved Japanese financial service companies. These companies were included in the sample frame in order to include companies outside of science, engineering and manufacturing, but without prior knowledge that the only projects these companies had with universities featured the universities as business clients. Several Japanese retailers were contacted for interviews, but they all declined saying they had no interactions with universities. It was surprising that few Japanese service companies seemed to think they could benefit from interactions with universities.

The two interactions that were principally training programs to benefit an industry (Table 1, 2^nd^ row) were in aerospace [Case S5] and IT communications. However, training was mentioned as a secondary objective of many research collaborations, particularly the large-scale (blue sky) Japanese ones [Cases S6]. Also the UK's KTP program, featured in several of the UK cases, is intended to mesh academic training with industry personnel needs.

## Findings Pertaining to Collaborative Research

### 1 Classification and Distribution of Collaborative Research Projects by Type of Company and Technical Field

The remainder of this paper analyzes the 80 collaborative research interactions, with the main focus being on IP and publication freedom. To provide a framework for this analysis, collaborative research cases are classified according to those involving:

1. startups (companies formed no later than 1990), sub-classified according to whether the startups are *spin-offs* from the university featured in the case.
2. other cases where the collaborative research is determined by the company and is intended to meet a fairly well defined need or to solve a fairly specific problem. These could be conceptualized as *applied research not involving startups*, and they are referred to as “typical” collaborative projects.
3. “blue-sky” research. This refers to collaborative projects where the companies are interested in how basic science can be useful for them, but where the solutions to the problems they hope to solve are unclear or complex, and where the applications of the research may be unclear. The companies involved in these collaborative projects were all large companies - most startups and small companies not being able to afford exploratory research with uncertain benefits. Although the company may define a particular area of research it is interested in, the specific research projects are often selected from proposals submitted by university scientists. Blue-sky projects are sub-classified as *light blue* and *deep blue*, with the former encompassing collaborations where the scope of research and the problems the company is trying to address are relatively defined, while the latter refers to projects that are more exploratory.

Table S1 lists all 97 companies featured in this study. The nine shaded in dark green did not describe collaborative projects. Five companies (including an unincorporated initiative) shaded in gray described collaborative projects, but were nevertheless excluded from the subsequent analysis [Note S4]. Table S1 also indicates which of the remaining 83 companies were engaged in startup, typical and blue-sky collaborations. Note that this number is higher than the number of collaborative projects shown in Table 1, for the reasons noted at the end of Methods, above.

**Figure 1:**
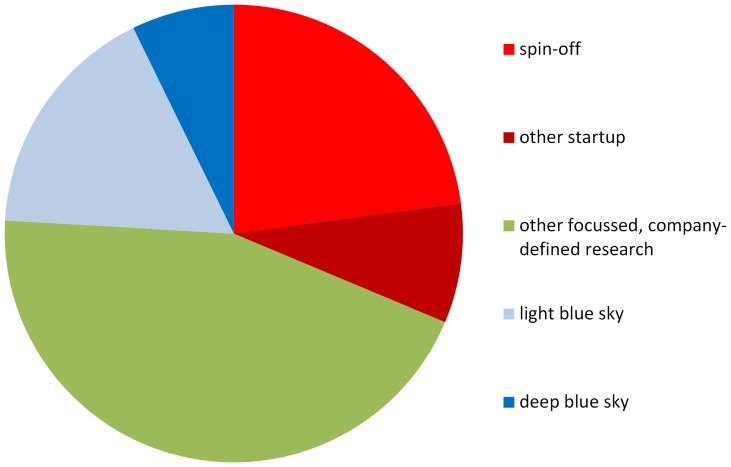
Distribution of the 83 collaborating companies according to the main type of research interaction that each described (from Table 2).

Table 2 shows the distribution of these 83 companies according to this classification, and Figure 1 shows this distribution graphically.

**Table 2:** Distribution of the 83 collaborating companies according to the main type of research interaction each described (from Table S1).

	Canada	Japan	UK	USA	total
spin-off	7	4	4	4	19
other startup	2	1	0	4	7
other focused, company-defined	5	5	19	8	37
light blue-sky	2	6	4	2	14
deep blue-sky	2	2	0	2	6
total # collaborating research companies	18	18	27	20	83

**Figure 2:**
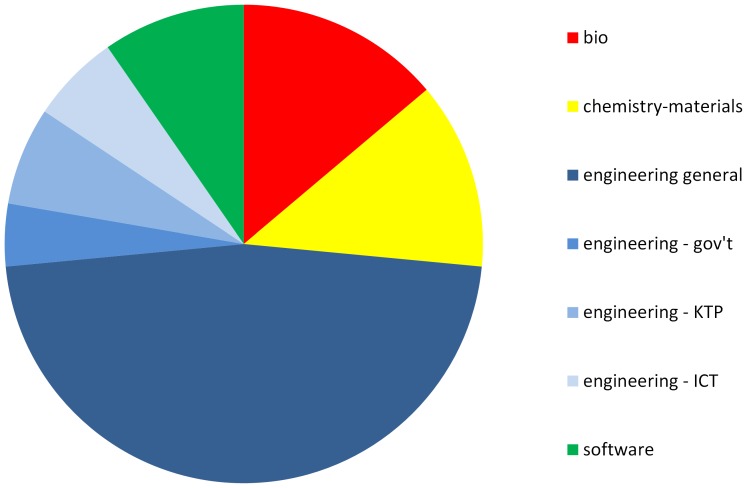
Distribution of the 83 collaborating companies according to the technical fields of the main research project(s) that each company described.

Figure 2 shows the distribution of these 83 collaborating companies according to the technical field of the collaboration highlighted in the interviews. The main categories are biology, chemistry and material science, engineering, and software. Within engineering, three special subcategories were created for projects focusing on information and telecommunications technology (ICT) [Note S5], projects under the UK's KTP program, and projects where a government agency is a major co-sponsor and will likely be the main customer (all of these are defense or aerospace related) [Note S6]. There were no ICT, KTP or defense-related collaborations that fell largely into software, biology or materials. If a project spanned two categories, the company was allocated half to each field [Note S7].

Not only were the vast majority of collaborations engineering related, but almost all the narrowly focused collaborations involving established companies (green in the previous Fig. 1) are engineering collaborations. This phenomenon is the same in all four countries. Keeping in mind the caveat about drawing inferences about rates from these data, this distribution is nevertheless striking, considering that university licensing income is generally highest from life science inventions and the largest numbers of spinoffs tend to be life science focused. However this is consistent with findings by Cohen and others that the direct impact of public science on industry most often occurs through engineering and applied science, especially materials and computer science [10]. It is also consistent with findings from Japan that engineering-related university discoveries are more likely to be developed by collaborative research than life science inventions [41].

As for the engineering collaborations with a defense or other government end user in mind, these all involved North American companies, mainly startups. This is another indication that the government's role as customer for innovative technologies is an important spur for engineering based entrepreneurship.

A majority of the biological collaborations involved startups, although large companies engaged in blue-sky research were also well represented. Hardly any biological collaborations involved established companies engaged in narrowly focused projects – probably reflecting the strong link between basic biomedical science research and the discovery of pharmaceuticals and therapeutic devices. A similar pattern emerges with the chemistry-materials collaborations. Most such collaborations are blue-sky projects involving large companies, startups come next, while focused collaborations with established companies seem to be relatively rare.

These distributions are relevant to the later discussion on IP management. The remainder of this section discusses findings from the collaborative research cases, beginning first with the collaborations involving startups, then the “typical” collaborations, and finally the blue-sky collaborations.

### 2 Startups as Collaborative Research Partners

#### 2.1 Insights into startups' role in an innovation ecosystem

Some of the collaborative projects described in the cases were projects that lead to the formation of the company or to its core technology. Others were projects initiated by startups after their formation. Not surprisingly, with one exception [Case S9], these projects are all defined by the startups' immediate development objectives. Nevertheless, some of the projects represent cutting edge science with potential for great impact, for example the research that lead to TeraView being spun off from the parent electronics company and Cambridge's Cavendish Laboratory [Case S7], and the research that lead to Transitive Technologies being spun off from the University of Manchester. Of note, large companies declined initial opportunities to develop these two groundbreaking technologies. TeraView's parent made a strategic decision that it was better to let a startup develop terahertz technology, while a large mainframe computer company turned down overtures from the inventor to develop what became Transitive Technologies' now widely used system to allow software compiled for a particular processor or operating system (OS) to be used on another processor or OS [Note S2].

Some of the other interviews suggested that large companies see healthy, independent startups as a vital part of a vibrant innovation ecosystem. Three of the large UK companies said startups were important in their industries to bring new technologies to proof of concept stage. One of these, a pharmaceutical company, said that it generally looked to startups, not universities, for new compounds. A large software company that began collaborating with a UK university laboratory on health applications of its software, found that the scientists employed by a spin-off from the same laboratory were quicker and more dedicated development partners than the university researchers [Case S3]. These case study observations are consistent with the finding that new companies are more likely than established pharmaceutical companies to undertake the initial development of innovative new drugs, especially those discovered in universities [42]. More generally, they are consistent with Christensen's observations how large companies often lose to new companies in new technology and business fields because of their natural tendency to focus on current successful high-income product lines and the needs of current large customers [43], [44].

Startups engaged in collaborations in all technology fields, but more engaged in engineering collaborations than any other – a finding that held across all countries. Bearing in mind the previously mentioned caveats, these findings suggest that the greatest need among startups for academic collaborations is in engineering related fields.

Almost all the startups described their collaborations with universities as successful. One of the common features of these collaborations is frequent contact between startup and university researchers. Sometimes they are one in the same. Even in the case of startups collaborating with another university, hiring of a student and frequent contacts with university researchers were cited as the most important mechanism of knowledge exchange and embedding. The Japanese interviews asked each respondent about number of person hours of face-to-face contact each month between company and university researchers. The average for the five Japanese startups was much higher than for the established companies, even for startups that were not spin-offs from the particular university [Note S8].

Some of the spin-offs remained based in the founder's lab for several years and this was also cited as a factor helping knowledge exchange and early growth. The duration was longest in the case of Canadian spin-offs. One American spin-off said that when the university suddenly decided it had to leave (probably because of conflict of interest concerns) the sudden expulsion “left a bad taste” and collaboration with the university ceased.

The startup interviews also suggest that academic entrepreneurship might be fostered by allowing more scientists to transition from industry to academic careers. Transitive Technologies and a Canadian spin-off developing a method to produce drugs and proteins from plants were both founded by university scientists who had done substantial research in industry before moving to academia [Case S8].

In the four years since the interviews, the overall business picture has been mixed. Some of the startups have substantially increased sales, received large private investments or been acquired on favorable terms. Others have not seen revenue gains or have failed. There are no patterns in our data that seemed to predict which companies would do well.

#### 2.2 Intellectual property and secrecy issues involving startups

Almost all of the startups indicated that patent protection covering their core technology was important. The most commonly cited reasons were: First, IP provided a degree of ownership and exclusive control over their discoveries, and defined those discoveries and control in a way that was important to attract alliance partners and investment. Second, they lacked other means, such as trade secrets and the ability to conduct large-scale development in-house, to prevent potential competitors from copying their discoveries. [Case S7, Case S8] This is consistent with other studies showing that startups in most industries, not only pharmaceuticals, need strong (in most cases, exclusive) IP rights covering their core technologies [45], [46]. (But compare [24]). One Canadian university appeared to have adopted a policy of letting all founders own their inventions, and one US university allowed the relationship to be structured as a contractor – contractee relationship allowing the startup to own IP rights. In only one or two cases was it difficult for the startup to obtain the IP rights it needed and in these cases problems were resolved as the parties came up the learning curve and became more flexible (for example [Case S8]).

Concerns about confidentiality and publication freedom were mentioned by several startups. One US startup said that, after the university finally came to understand the research interests of the company (adhesives under extreme conditions) it was able to work out an agreement that included academic publication rights but also rights of the company to limit data release. (The transcript did not indicate whether the company's right to limit data release was limited to preventing publication of the startup's own trade secrets and ensuring a few months to prepare patent applications, or whether the company sought broader authority to limit publications.) Ultimately the company was pleased with the collaboration outcome, noting that it planned to publish papers on the results and that these results benefitted both its near and long-term business goals. Another US startup also praised its association with a nearby university, but noted tensions related to publication freedom and said that it structured its interactions with faculty as consultations, in part to avoid tensions over publication issues [Case S9]. A Canadian startup had the university sign a non-disclosure agreement, although it ultimately decided that the PhD student, whose project was the core of the collaboration, probably could publish what he wished. A Japanese startup said that it did occasionally exercise its standard contractual right to review academic sponsored research publications and to require changes.

### 3 “Typical” (i.e., focused, company-defined) Collaborative Research (not involving startups)

This was the largest category of collaborations, with 36 companies - over half from the UK. All except five or six were engineering related. These “typical” collaborations involved companies ranging from a 23-employee company making specialty textile products to IBM. The IBM project involved collaboration with a UK university to establish a nationwide mammogram database. Another large project involved Advantest and the University of Tokyo establishing a new on-campus facility for research and education on the architecture, manufacturing and testing of VLSI chips [Case S10]. Arguably, this project could be classified as blue-sky.

Two of the smallest companies said they could not afford to employ more than a few university graduates. [Cases S11] summarizes these two cases and how the companies engaged universities at the margins of their absorptive capacities. None of these cases raised IP or publication freedom issues.

Aside from these smallest companies, the companies' retrospective assessments did not indicate absorptive capacity was a problem. Most companies cited concrete benefits. Furthermore, some of the large companies with strong internal R&D capabilities, said that these collaborations expanded their perspectives on scientific and engineering issues in ways they often did not expect [Case S3, Case S10, Cases S12].

In order to make sense of the large number of cases, the analysis focuses on IP and confidentiality issues country by country.

#### 3.1 Canadian “typical” collaborations

The five established Canadian companies engaged in focused collaborations did not have complaints about IP matters. One said that, in view of it not having paid full project costs, it was satisfied with the right of first refusal to negotiate a license. One indicated it secured satisfactory rights to an invention. Three said that no IP emerged. All of these collaborations were related to mechanical engineering.

However, two companies did raise confidentiality concerns. One said that non-disclosure agreements (NDAs) are the most important legal agreements when dealing with universities [Cases S13].

#### 3.2 Japanese “typical” collaborations

IP conflicts were rare in the Japanese cases because, as described below, Japanese companies usually end up with exclusive control over collaborative inventions. As also discussed below, standard Japanese collaborative research contracts allow companies to restrict publication of collaborative research findings, and about half the Japanese companies said they sometimes exercise this right. However, one company explicitly stated it never exercised this right and always supported academic freedom to publish [Case S14].

#### 3.3 UK “typical” collaborations

About half the UK companies said IP was not an issue and that IP was not expected to arise from the collaborations. However the great majority of these companies were engaged in Knowledge Transfer Partnerships (KTPs) with universities [Note S9] [Cases S11, Cases S15] or were large corporations engaged in software or data management collaborations [Case S3, Cases S12].

Among the ten collaborations in which the company considered IP to be important, four cited problems related to university IP management. The most frequent was that universities overvalued their IP, thus making negotiations difficult [Cases S16]. One company that did not encounter problems in the collaboration it described, nevertheless noted a trend towards stricter IP management by universities motivated by the goal of making money.

However, half of the ten companies seemed to be satisfied with the way IP issues were managed and indicated they obtained the IP rights they needed, whether exclusive or non-exclusive. One of these accepted that it that would not be able to own collaborative inventions, but would at least have freedom to use them. Perhaps this reflects a relatively common understanding between companies and universities based upon the Lambert Model Agreements, discussed below.

Problems relating to confidentiality were not mentioned, perhaps reflecting a common understanding about publication freedom based upon the Lambert Model agreements.

#### 3.4 US “typical” collaborations

Concerns about IP and confidentiality were mentioned more frequently in the “typical” US cases than in those of the other countries. All of the eight US companies involved in these focused collaborations mentioned IP-related tensions. In contrast to the complaints from the UK respondents that focused on naïve greed or incompetence, the American complaints more often dealt with fundamental issues, such as companies' ability to obtain exclusive IP rights and their need to keep some of the research results confidential. Although the most frequently articulated concerns dealt with increasing university assertiveness over IP rights and having to pay twice for research, the deeper underlying concerns often seemed to involve confidentiality issues and how to prevent competitors from accessing potentially strategically important information and technologies arising from the collaborations. These findings coincide with observations from Pennsylvania State University that, although companies may often begin a list of complaints with the double payment issue, their underlying concern most often concerns the possibility that they will lose control over important technologies arising from collaborative research [20]. The following is a summary of each of the eight “typical” US cases, focusing on the IP and publication aspects.

1. *A case showing sensitivity about publishing collaborative research findings*: In a case involving engineering design and consulting, the negotiations between the company and university centered on the degree to which information resulting from the project could be made public and accessible. The company wanted all of the project details to remain private, while the university wanted to ensure its researchers could publish their results. They compromised with an agreement to allow the company to review any publications prior to journal or conference submission. The main legal transaction that took place was an NDA. Once the project was completed, a license was concluded that gave the company exclusive rights relative to its specific business field, but allowed the university to license outside of the company's main market. The respondent was an area manager for the company and was also responsible for its entire intellectual asset management operations.

2. A case showing how university policies evolved to take into account the company's concerns about its ability to use (and to prevent competitors from using) university discoveries: The respondent was General Manager of a company that collaborated with a nearby university on two projects, one on ways to make its manufacturing processes safer, and another on analytical testing of its products. The first collaboration was nearly stillborn when the university insisted on owning any emerging IP and also charging the company for its use (i.e., for a non-exclusive license). The company felt it would be paying twice for the research and that competitors would have equal access to the research findings. After discussions, the university changed its policy. In the case of the second collaboration which was subject to the new policy, the company was promised a royalty free license to any emerging technologies and there were provisions to limit competitors access, about which the respondent did not provide details. The company also felt assured by the realization that significant IP would probably not emerge from these collaborations.

3. *A case showing concerns about confidentiality and universities overvaluing their IP:* The respondent, a vice president for an engineering company, said the company values relationships with universities primarily for recruiting. Collaborative projects arise usually through personal contacts between company and university researchers and are motivated by needs of particular projects the company is working on. (I.e., joint research projects usually arise as subcontracted projects under a larger contract with a third party.) The company acknowledged tension between the university culture of openness and a competitive business environment that pushes companies to try to obtain competitive advantage either through proprietary positions (e.g., patent ownership or exclusive licenses) or unique capabilities (e.g., trade secrets). In order to try to bridge these differences, the company worked out blanket joint research agreements with the three regional universities with which the company works most closely and from which it recruits most frequently. However, these negotiations were not easy, especially for the first agreement, which took 1.5 years. If there had not been champions on both sides, the negotiations would have failed. Even after the blanket agreements were concluded, agreeing on licensing terms for specific collaborative inventions proved difficult. The most common problem was universities overvaluing their IP and underestimating the costs and risks of further development by the company. The respondent remarked that it is still rare for the company to take university (or joint research) IP all the way to market commercialization.

4. *A case showing unease with respect to increasing university assertiveness over IP rights*: A manufacturing company described what are essentially student internship programs under which the students spend one to three months working in company facilities on projects that address real challenges faced by the company. The first was set up with an internationally known university through the company's membership in one of that university's industry liaison programs. The second was an adaptation of the first program to enable the company to work with students and faculty from a well-known nearby university. The respondent, the company's Chief Scientific Officer, praised the results of both programs, describing examples of how they had made manufacturing more efficient and had helped to keep company R&D staff up to date with current knowledge. The respondent also praised the administrative simplicity of the programs. Hardly any contract negotiations were involved. Students signed a confidentiality agreement with the company. If any IP is generated, the company will own it. However, at the end of the interview, the respondent offered cautionary observations that the IP management practices of US universities are creating significant stumbling blocks to enhancing industry-university cooperation. The respondent suggested that the most troublesome university practices stem from their concern that they (the universities) not give away valuable IP. The respondent also suggested that membership fees in university industry liaison programs are becoming too high – and companies are being forced to question whether the costs justify the benefits. The respondent urged universities to approach IP ownership/control issues pragmatically, taking into account who defined the research problem, who developed the solution, and who paid for the solution, sentiments also echoed by the case highlighted in [Case S9].

5. A case showing concern about publication rights and IP, but also how experience has helped both sides work together more smoothly: The Director of University Programs for a manufacturing company described how it has begun to focus its university interactions on a small number of strategic university partners – a strategy that is being followed by other large companies. In the case of the particular university under discussion, the respondent said that the university's office of corporate research has been extremely helpful in matching company needs with university research and in coordinating interactions with the technology transfer office. The respondent noted that “In the past, the sticking point was publication rights. Now the company and its strategic partners are able to compromise and consider when and what to publish, such that the university gains publication rights yet the company addresses its confidentiality concerns (details not provided). More recently IP rights have become the sticking point. Universities are now more protective of their IP rights.”

However, the respondent also noted how the company and its university partners have learned to work more effectively together. The company has attorneys who now specialize in university transactions, and deals that fell through several years ago can now be concluded in 30 minutes. The respondent remarked that the company's sensitivity towards IP issues and need for guaranteed licensing rights vary according to the degree of direct impact of the research on the company's business and how close a potential product is to launch. The easiest negotiations involve collaborations based on fundamental research or that build upon on the university's research. The hardest negotiations concern projects where the company already has significant IP and/or is close to product launch.

*6. The most negative interview among all 90 raised concerns about IP, confidentiality, costs and bureaucracy:* The respondent, whose remit covers open innovation, technology scouting and external collaborations for a manufacturing company, spoke in general terms without focusing on a particular interaction or university. To paraphrase:

US universities are very difficult to work with. First, university overhead rates make them expensive and non-competitive. Second, the negotiations required for even a minor project can outweigh the benefits realized.

The most difficult item to negotiate is ownership of the IP. The company does not want to have to pay twice, once for the research then for a license. Typically three agreements must be negotiated just to get project started: 1) confidentiality agreements, sometimes with multiple parties, 2) a research contract which involves negotiation of research fees, overhead rates, and IP ownership; then 3) a license option covering future-developed technology. These three documents can take months to complete, slowing down the entire process.

In addition, the company often desires university partners that can model complex production scenarios and have equipment to test products or processes in such simulated environments. Many universities do not have such capabilities. In negotiations with potential US university partners, a typical response is “We don't have the right equipment, so pay us to get it.” In contrast, the company has found collaboration networks of universities in Canada and Europe that promote pooling of equipment and expertise among several universities. The company has experienced greater benefit and a much more productive attitude in working within these collaborative networks. The company is not aware of similar networks among US universities. It is also looking to developing countries with research infrastructure, such as India and China, because it is very affordable to do research there.

Another challenge is how much information to share with the university partner. The company must be careful not to divulge too much to university staff, especially students who may take that information with them anywhere, including when they go to work for competitors. Confidential agreements are signed with the university, not with an individual student, which increases risk of dissemination of proprietary information. The company requires time to review any presentation or manuscript in advance to ensure no proprietary information is disclosed and patentable inventions are properly protected. The company does not want its name to appear in a paper or presentation without prior written permission. *(Comment: At one level, these confidentiality expectations are commonplace in collaborative research. However, the interview suggests that most of this company's collaborations involve production processes, which are harder to protect with patents than composition of matter inventions. Also, if the background knowledge that the company brings to the collaboration consists mainly of production process knowledge (often protected as trade secrets), and the research itself deals with production processes, it may be hard to disentangle proprietary information that the company brought to the collaboration and academic research findings. Furthermore, it may be challenging to shield the company's background proprietary manufacturing knowledge from disclosure in academic publications even when only findings made in the academic laboratory are published. While this respondent did not explicitly say that the company's pre-publication review to prevent disclosure of proprietary information might lead to suppression of academic research findings, some of the Japanese interviews explicitly acknowledged that this does occur.)*

7. This case shows how a company addresses its need to control any IP emerging from university interactions by structuring such interactions as consultancies: The company's focus is on software development for manufacturing operations within the larger multinational corporate structure. It relies on university professors mainly to help conceptualize very early stage technologies, but develops prototype software and does all further development in-house, thereby helping to ensure that it owns all relevant IP. Student interns are used extensively. Since they work inside the company, this also ensures the company will control emerging IP. Sometimes it will hire students as employees “in order to avoid IP issues.” The respondent, the CEO, said that for ten years the company had not conducted any sponsored research in universities. Rather it structures interactions with faculty as consultancies so that it can own any resulting IP, as in [Case S9]. Its general principle is that it will not pay for IP that it will not own.

Since research is focused on software and mathematics, the company can generally avoid significant use of university research facilities. If a university asserts ownership over an invention by a consulting faculty member, the company will offer to pay patent prosecution costs in exchange for ownership. If the university insists on sharing in any revenue stream, the company has a formula to accommodate such demands. The respondent remarked that, “Overall, most professors find that consulting is a better arrangement than our sending them funding through the university.”

8. This final case shows another example of a company that wants to own IP emerging from collaborations, but is willing to settle for an exclusive license. The respondent, a research director for a manufacturing company did not offer any specifics about projects or universities. The interview was notable for the observation that if the company pays for research, it wants to own it. However, if the university refuses, it may settle for an exclusive license for a specific field of use or time period.

### 4 Blue-sky Collaborations

#### 4.1 General observations

Blue-sky collaborations were divided roughly equally between engineering, chemicals-materials, and biology.

Both light and deep blue-sky collaborating companies spoke of the benefits of new perspectives and new ways of thinking. Most companies engaged in light blue projects cited concrete benefits. About half of these companies said the benefits were linked to a long-term relationship with a particular professor. For example, one materials science company suggested that it owed much of the scientific underpinnings of one of its energy-related initiatives to a particular professor's laboratory. However, some of the companies engaged in deep blue-sky projects (most of which were in pharmaceuticals or chemistry) had difficulty pointing to tangible benefits. Despite management having granted these projects long time horizons, the interviews suggested that most deep blue-sky projects eventually must show concrete benefits, and some of the project managers are concerned this time may come sooner than they hope [Cases S17].

#### 4.2 IP and confidentiality issues

In contrast to the pervasive disagreements characterizing the initial stages of focused US collaborations, interviews with US companies concerning blue-sky collaborations registered the lowest frequency of dissatisfaction, aside from the Japanese companies who almost automatically have a privileged IP position. However, as the number of respondents was small, comparisons must be made with caution. The single US company expressing dissatisfaction said that, when it sponsors research in non-US universities, it does not let them have any IP rights, and thus it does not make sense to have to pay twice for research results from American universities. But it added that sponsors' default IP rights should be a free right to use IP arising from the collaborative research, implicitly acknowledging that it might be reasonable for the university to charge royalties for an exclusive license. Two American companies that described blue-sky collaborations said they were comfortable with university IP ownership provided they had the first option to negotiate for an exclusive license [Case S18].

An American pharmaceutical company described deep blue collaborations where it did not even need this right. If its own scientists determined, after confirmatory in-house research, that a collaborative discovery was commercially important, it would then approach the university to negotiate a license, realizing that the university was not obligated to license to it [Case S19]. The respondent acknowledged that, if the company were to control collaborative discoveries without development obligations or incentives, it would tend to treat them as free goods and would be less likely to devote resources to develop the drug and biomedical device candidates in-house. Also he acknowledged that startups backed by venture capital often develop early stage discoveries faster than large companies.

In contrast, at least two of the four Canadian companies said they preferred to own blue-sky collaborative research inventions, although they were encountering situations where ownership was becoming more difficult. One company that did not express a desire for ownership nevertheless cited problems with universities overvaluing early stage inventions and not appreciating the effort necessary for the company to develop them.

Two of the four UK companies had complaints regarding the Lambert Agreement principle that, if they are to own collaborative research inventions, they ought to pay the full economic cost (FEC) of the research. Both objected to paying FEC. One echoed the complaints of companies engaged in “typical” collaborations that UK universities often overvalue IP and do not understand the effort required to create value from university inventions — adding that IP issues de-motivate company collaborators [Case S20]. The other company said that it was restructuring collaborations as consultancies in the expectation this would give it automatic control over emerging IP [Note S11]. A third UK company did not have complaints, but it ended up controlling the main technology at issue [Case S7]. The fourth company said university patents are usually too narrow and easy to invent around, and thus it rarely licenses them.

Why this apparent difference between the situation in Canada and the UK on the one hand, and the USA on the other? Some of respondents mentioned that their blue-sky collaborations were often preceded by more focused collaborations. This enabled their companies to become familiar with IP management policies of US universities. These policies generally include:

- university ownership of IP,
- no pre-negotiated licenses before inventions arise,
- the requirement that any licenses (even non-exclusive) be negotiated,
- royalty rates that some companies consider to be high but which are, in any case, subject to negotiation, and
- requiring exclusive license contracts to include development obligations and other development incentives. These might include obligations to meet specific development milestones or to pay substantial royalties (sometimes on an annual basis) — although most universities are open to re-negotiating these obligations if the company is proceeding with development but is encountering difficulties.

But simply being familiar with such policies would be unlikely to make companies acquiesce to them without complaint, considering that the companies involved in typical (focused) collaborations readily expressed dissatisfaction with the same policies.

Instead, probably the companies find these policies less threatening in the context of exploratory blue-sky research. Only about half of the blue-sky respondents mentioned specific university discoveries (including analytical tools, as opposed to actual products) that were important outcomes of the projects [Note S12]. Many suggested that companies perceive that intangible benefits, such as new perspectives and exposure to frontier areas of science and engineering, are more important than rights to patents covering early stage discoveries. [Case S19] and the interview summarized in “typical” American case no. 5, above reflect this perspective. They also suggest that a frequent corporate approach to blue-sky university discoveries is to assess the university discoveries in their corporate laboratories. If the companies elect to go forward with development, they will likely be able to control the more commercially important downstream inventions. One UK company described important inventions in commercial use made by its own scientists whose knowledge base had been enhanced by blue-sky collaborations with university laboratories.

Companies also seemed to accept academic norms on publication freedom more readily than in the case of focused collaborations. None of the Canadian, UK or US companies engaged in blue-sky research mentioned the need for NDAs. One UK company said it had the right to delay publications up to 9 months, but it has never exercised this right. Perhaps this lower concern with secrecy in the case of blue-sky collaborations reflects some of the factors mentioned above, or perhaps greater university insistence that research that is close to fundamental research be freely publishable. The next section discusses this issue in more depth.

The experience of university technology management offices may also explain in part the differing corporate perspectives on typical versus with blue-sky collaborations. In the North American cases, most of the frustrations arose during “typical” collaborations with universities that had small or recently established technology management offices. Among the five North American blue-sky collaborations that focused on a particular university, two involved an internationally known US university with a highly regarded technology management office. The respondents in these cases praised the professionalism of this office and the ease of working out mutually satisfactory IP arrangements, even though this office is known for upholding university interests and publication freedom. These assessments about the advantages of working with experienced technology management offices are echoed in “typical” American cases nos. 2 and 5, above and also in interviews with companies outside of this study [Note S13]. At least in the US, blue-sky collaborations may be more likely to occur in universities that have both industry-relevant scientific expertise, and the ability to manage IP and publication issues smoothly.

In contrast to the American companies, all but one of the eight Japanese companies engaged in blue-sky collaborations said they expect to have exclusive rights over collaborative discoveries. Also three of these eight companies said they do restrict publications while three more said they might occasionally exercise this right – a right guaranteed under standard collaborative research contracts [Note S14].

## Discussion – National Systems for Managing Collaborative Research

The Japanese situation presents an interesting contrast to that in the other three countries with respect to both the pervasiveness of collaborative research and the degree of control corporate collaborators have over IP and publications.

Joint industry-university patent applications, most of which cover collaborative research inventions, are the dominant form of technology transfer in Japan, vastly exceeding licenses of independently invented university discoveries. Since 2007, such joint applications have accounted for about 60 percent of total university patent applications. Because only a fraction of the remaining 40 percent of university applications are licensed, the ratio of jointly patented collaborative research inventions to inventions transferred under independent licenses is roughly 3:1 in major universities and probably even higher in lesser known universities. The vast majority of the industry co-owners are large Japanese companies. [47]–[50]

At the end of the application process when patents issue in various countries, over one-third (perhaps over half) of patents issued to Japanese universities are co-owned by companies – the overwhelming majority of which are large Japanese manufacturers [Note S15]. This situation is probably unique among major industrialized countries. Only about 15 percent of German university inventions that have been awarded US patents are co-owned by companies. For Canadian and UK university inventions, the proportions are about 10 and 6 percent, respectively [48], [49] [Note S15].

The percentage of US university patents that are co-owned by companies is less than five. This is due in part to a unique aspect of US patent law under which patent co-owners can transfer their rights without the permission of other co-owners, thus making patent co-ownership of a US patent equivalent to a transferable, royalty-free, *non-exclusive* license. In all other industrialized countries, the permission of all co-owners is necessary for any license or assignment, and thus co-ownership is equivalent to a non-transferable, royalty-free, *exclusive license* [50]–[52].

In any case, having companies lined up as development partners for such a large proportion of Japanese university inventions may provide a quick and non-bureaucratic route to commercialization. This may be particularly valuable when university technology management offices do not have the skill to manage many of their inventions.

However, with co-ownership comes automatic control without development obligations. Among the approximately 175 US patents issued jointly to Japanese universities and companies within a twelve month ending in mid 2012 [Note S15] about half either are broad patents potentially useful to several companies in the same industry, or represent potentially significant technical advances with important market impact. If the company co-owners of these technologies do not try to develop them, or do not at least seriously look into the feasibility of development, this would represent a loss of potential benefits to society. It would also signify loss of the taxpayer funding for the university research that lead to these inventions. The amounts that companies pay for joint research in Japanese universities usually cover only a fraction of research costs. Furthermore, the overall proportion of university research that is supported by industry is much lower in Japan than in most other large industrialized countries with a strong science and technology base [Note S16].

The interviews indicated that many collaborative discoveries are not developed. The Japanese respondents often said that moving collaborative discoveries from the corporate basic research laboratory to the development laboratories is problematic. The research managers who champion university discoveries in corporate basic research laboratories often are not able to convince senior management to move the projects out of the basic research laboratory and into development phase. The respondents for most of the blue-sky collaborations noted that while some university knowledge is embedded in the company, it is rare for commercial products or processes to evolve directly from university collaborations.

Large Japanese companies may be no different than other large companies in this regard. However, because such a large proportion of Japanese university discoveries are co-owned (and thus essentially locked up) by large companies without any development obligations, the risk associated with sponsors having automatic co-ownership of inventions is great in Japan – especially in the case of blue-sky inventions.

One of the greatest dangers of lock-up, at least in Japan's case, may be the foreclosing of opportunities for startups to develop promising university discoveries. Separate studies have documented large Japanese companies saying that it makes sense to co-patent upstream university discoveries because this will pre-empt their use by competitors. These companies also add that a startup whose key IP is compromised by co-ownership by a large company would usually be disqualified as a potential investment target [47][Note S17].

A 2008 METI white paper showed that most large Japanese manufacturers have no system to support the formation of spin-offs or even to launch internal corporate ventures to develop idle technologies. 60 percent of Japanese manufacturers said that whatever technologies they do not commercialize themselves are simply abandoned and are never made available to outside parties. Furthermore the white paper noted that, as competitive pressures and financial constraints increase, companies are increasingly reluctant to develop any technologies not related to their core business [53]. These constraints were probably more severe at the end of 2013 than when the companies were surveyed in 2007.

The accounts of the formation of TeraView and Transitive Technologies [Note S2] parallel closely Christiansen's description of how large companies often fail to develop new technologies. Common reasons include not perceiving their value, thinking the market is too small, considering the technologies too removed from their main business or main customer needs, or being too bureaucratic to develop them rapidly [43], [44]. Henderson [54] describes how normal decision-making processes in large companies often mitigate against a concerted effort to develop new technologies. The only unusual aspect of the TeraView case was that the parent company agreed to let TeraView have exclusive rights to the IP it needed to launch its business [Case S7].

UK and most Canadian universities have taken steps that prevent automatic lock-up of collaborative research inventions – particularly those with industry and university co-inventors that would otherwise be automatically co-owned and thus exclusively controlled by the companies. The UK has done so through the Lambert Model Agreements, which were formulated by representatives of industry and universities in 2003-2004 and began to be broadly used by 2008 [note S18]. The five model agreements are voluntary. However, they embody the principle that universities and companies should decide in advance what rights the company will have over resulting IP and publications. Agreements 4 and 5 give the company automatic ownership rights over IP, with agreement 5 giving the company the right to control over publication. (All the other model agreements uphold the right to publish after the company has had several months to review manuscripts to ensure that none of its own confidential information is divulged and to prepare patent applications.) However, if the company is to own the IP, it should pay at least the full economic cost (FEC) of the collaboration, including attributable portions of the salaries and stipends of university researchers and staff, and all other direct and indirect costs, including infrastructure costs and depreciation. As noted above, some of the UK companies engaged in blue-sky collaborations complained about the requirement to pay FEC. Agreement 3 gives the company the right to negotiate for assignments (including to limited fields of use) after inventions arise. Agreement 2 gives the right to negotiate for exclusive licenses. Agreement 1 simply grants an automatic, royalty-free, non-transferable, non-exclusive license – a right also automatically granted in Agreements 2 and 3. Embodied in Agreements 2 and 3 are the principles that royalty payments should reflect that value of the IP rights obtained (“the University is obliged to seek a fair return”) and that the university can require transfer of rights back to the university if the company is not meeting development or royalty payment obligations built into the licensing or assignment agreements.

It should be noted that under the UK Patents Act of 1977, section 39(1), inventions made by employees in their normal course of work belong to their employers. Most UK universities rely on these default provisions to assert ownership over inventions by their faculty – especially in the case of collaborative research inventions arising under contracts between companies and the universities [51]. The authors know of no UK university where this is not the case. Therefore the Lambert Model Agreements are probably applicable to all university-industry research collaborations in the UK.

Canadian universities are, in the words of one company respondent, “all over the map” with respect to IP policy. However, most research-oriented Canadian universities assert control over collaborative research inventions arising from moderate to large-size projects and require that corporate sponsors negotiate with the universities for IP rights. The University of British Columbia has essentially adopted a simplified version of the Lambert system, offering sponsors the option of choosing between Lambert Models 4 (ownership conditioned upon payment of FEC), 2 (option to negotiate an exclusive license) or 1 (automatic royalty-free non-exclusive license). All of these options ensure freedom of publication, allowing delays of no more than six months to ensure that company trade secrets are not divulged and to file patent applications [Note S19]. The University of Toronto permits joint ownership in the case of university and company joint inventors, but then gives the company 60 days to decide whether it wants to license the University's rights, otherwise the University is free to license its rights to a third party. Publications can be delayed for no more than 90 days, and the University can insist that there be no delay at all for evaluation of graduation theses [Note S19]. McGill University's policy is that “commercial rights to the arising intellectual property are negotiated, and can vary depending on the nature of the collaboration and the contributions of both parties [Note S19].” The most notable exception is Waterloo University, which maintains its policy that inventors own their inventions and decide their disposition, even in the case of joint research inventions [Note S19]. Also Queens University generally lets its researchers retain ownership over their inventions. However, if a corporate research sponsor insists that inventions be owned by the university or the company, then Queens University can insist on such a transfer [Note S19]. Other Canadian universities that otherwise permit inventors to retain ownership of IP, usually require that they assign their rights over sponsored research inventions to their universities [Note S19].

Against this background of varying policies, the Natural Sciences and Engineering Research Council launched the Engage Grants program in 2010. This provides up to $25,000 to Canadian university researchers for focused, short-term (up to six months) collaborative R&D projects with companies, and requires that any IP arising from the projects be owned by the companies [Note S19]. As a result of this program, many universities are reconsidering the importance of retaining IP rights arising from collaborative research.

The effect of the UK Lambert Agreements and the IP management procedures of most Canadian universities is to supersede the default provisions of UK and Canadian patent laws. These would otherwise result in co-ownership of co-invented collaborative research inventions. Such co-ownership would otherwise enable the companies to lock up these inventions without facing any development obligations, by virtue of the patent laws requiring that all co-owners must agree to any transfer, but allowing each co-owner free use of the inventions. By giving universities authority to negotiate issues such as ownership, license terms, and development obligations at the outset of a collaboration (or as inventions arise) regardless of whether the inventors are all university researchers or a combination of university and company researchers, UK universities and most Canadian universities pre-empt the sponsors insisting that, as a general principle, they should own or co-own all sponsored research inventions and thus completely control them. They also pre-empt automatic company co-ownership and control simply by virtue of a company researcher being named as a co-inventor – which is the usual fate of Japanese collaborative research inventions.

Since 2004, Japan's state supported universities, which are its major academic research centers, have had independent administrative status. (They are officially called *national university corporations,* and in 2011 they numbered 86.) Obtaining independent administrative status meant that, under Japan's Patent Law article 35, the universities, as employers, can require their faculty, as employees, to assign their inventions to the universities. Acting under guidance documents from the government, they generally do require assignment of patentable staff inventions [56] [Note S20]. Thus in terms of the basic ownership structure of university inventions and also the default patent law provisions regarding rights of co-owners, Japanese universities are similar to most UK and Canadian universities.

However, unlike their UK and Canadian counterparts, most Japanese universities do not try to unify IP rights covering collaborative research inventions under their control, which would enable them to negotiate transfer terms with the research sponsors. Instead, they simply let the default provisions of Japan's patent law govern ownership and control. Company employees are almost always named as co-inventors along with university researchers as a result of discussions between the academic and company scientists leading the projects. Thus the university and company end up as co-applicants and subsequently as co-owners if patents issue.

The main exception is the University of Tokyo whose model contract contains a clause (article 21) that attempts to override the default provisions of Japan's patent law by stating that the University of Tokyo can license its rights to a third party unless the company negotiates an exclusive license to the University's rights *or the company opposes the transfer with reason*. Of course, this last qualification permits companies to stand firm and block such transfers [48]. The University of Tokyo was the one university singled out for criticism by several of the Japanese respondents for being uncooperative with respect to contractual and IP matters. Nevertheless, it has continued to try to require collaborating companies to negotiate for exclusive rights and to require that the resulting license contracts contain develop incentives.

Recently, a major Japanese university has succeeded in convincing several pharmaceutical companies to agree to a limited period of exclusive license rights for drug candidates arising under collaborative research. If the companies desire exclusivity beyond this period (for example, beyond three years), they must demonstrate they are moving forward with development.

In the United States, the right of universities to own faculty inventions is based upon the common law right of employers to require employees to assign work related inventions to their employers. The precedents that set forth the actual conditions under which universities can require faculty members to assign their inventions to their universities date back to negotiations between MIT and its faculty in the 1930s [57]. However until 1980, the contracts and grant agreements under which US federal government agencies funded university research generally required that the funding agencies own emerging inventions. Because federal support accounted for about two-thirds of all US university research funding in the 1970s, most US university inventions were government owned. Furthermore, only after 1971 did federal agencies have authority to issue exclusive licenses to companies interested in developing such inventions, and this process was cumbersome [58], [50], [55].

Thus, on the surface, the American and Japanese systems were similar until 1980 [Note S20]. However, compared to Japan, there was a longer tradition of university ownership of non-government-funded inventions and stricter government agency monitoring of government funded inventions. The practices of inventors attributing government-funded inventions to other funding sources, and universities and government agencies turning a blind eye to direct transfers of IP rights from university inventors to companies were less common in the US than Japan.

Thus, pressure in the US began to build in the 1970s to permit the exclusive licensing of more federally funded university inventions, particularly those in pharmaceuticals and other chemicals. This pressure, combined with concern that American competiveness was suffering because of weak incentives to develop university discoveries, lead to passage of the 1980 Bayh-Dole amendments to US Patent Law. This Bayh-Dole law (Public Law 96-517, codified at 35 USC §§ 200-212, with implementing regulations at 37 CFR §401) permitted universities to own federally funded inventions and, under certain conditions, to license these exclusively to companies [58]. However, assignment (sale, transfer of ownership) of federally funded inventions was prohibited absent permission from the funding agencies. The Bayh-Dole law also imposed on universities strict requirements to report to the funding agencies steps the universities were taking to commercialize these inventions.

Largely because of the need to implement the reporting requirements, most universities that previously had permitted faculty to retain ownership over non-federally funded inventions began to require that all faculty inventions be assigned to the universities [59]. This move to assert control over all faculty IP also extended to inventions arising under industry-sponsored research. However, a notable difference between the US and the other three countries is that co-inventorship does not offer companies an automatic lock on inventions as it still does in Japan, and as it would in the UK and Canada absent the IP management provisions in those countries discussed above. Co-ownership of US university patents is not as valuable for industry research sponsors as it is in other countries, particularly Japan. This probably explains why the rate of industry co-ownership of US university patents is less than five percent. This is lower than industry co-ownership rates of UK, Canadian and German university patents, and considerably lower than the co-ownership rate of Japanese university patents [Note S15].

Recently some American universities have retreated from asserting control over sponsored research inventions. In December 2011, Pennsylvania State University announced that it will no longer require that sponsored research inventions be owned by the University. That same month, the University of Minnesota announced that if corporate research sponsors pay an additional 10 percent of the contract cost, they can have royalty free worldwide rights to the inventions arising under their sponsorship — except that the sponsor will pay a 1 percent royalty on annual sales exceeding $20 million. Penn State has also incorporated this “home run clause” into its standard sponsored research contract. These two universities made these changes because disputes over IP rights were taking too long to resolve and were resulting in significant lost sponsored research opportunities, and also because licensing revenue from sponsored research inventions is usually low [60] [Note S21].

## Conclusions

### 1 Key Findings

For the sake of clarity, the following list summarizes the main findings from this study:

1. Dissatisfaction with university policies preventing companies from automatically owning (or otherwise controlling) collaborative inventions was most acute in the case of narrowly focused (typical) collaborations, particularly those with American universities but also, to perhaps a lesser degree, with UK universities. However, in the case of blue-sky collaborations, the country patterns were reversed with few complaints directed at American universities, but dissatisfaction frequently voiced related to collaborations with UK and Canadian universities.

2. Japanese researchers in corporate, basic-research laboratories work closely with their university counterparts. However, it is often difficult to gain management support to move university-inspired discoveries to development/commercialization stage. The fact that the collaborating companies exclusively control the university discoveries, generally are under no obligation to pay the universities royalties, and have wide discretion to limit the university researchers from publishing their findings, raises the question whether such broad corporate control over collaborative research results might be a disincentive for the companies to invest in developing the university discoveries.

3. Despite guidelines and policies upholding publication freedom for university researchers engaged in industry-sponsored research, publication freedom remains a contentious issue in some collaborations. However these collaborations seem to involve mainly startups (which have to reveal their core technologies to university collaborators), large companies engaged in engineering collaborations (particularly related to manufacturing technologies), and collaborations with North American universities. It is tempting to speculate that the low incidence of publication freedom disputes involving UK universities is due to the Lambert Model Agreements having established universally accepted ground rules - including an option that allows companies to limit publication freedom if they pay full research costs.

4. Some UK companies resent the obligation under the Lambert Agreements that they pay full research costs (full economic cost, FEC) in order to have the right to own emerging IP or to limit publications.

### 2 Suggestions Regarding IP Management

Amidst the varying approaches to management of IP and publication rights in the context of collaborative research, the case studies, and in particular the key findings above, suggest some steps that may provide a mutually satisfactory middle ground in most situations.

At the outset of a collaboration, it may be useful to distinguish whether the research project is focused or blue-sky in nature.

In the case of a focused project largely determined by the company, the university stepping back and letting the company have exclusive rights (e.g., granting the company a royalty-free exclusive license or perhaps even outright ownership) would eliminate much of the frustration expressed by companies. In such projects, the risk of the company locking up, but not developing, valuable discoveries is probably small, because these projects are oriented to clear, specific, near-term business needs. One way to mitigate this possibility would be to require companies that want exclusive control to pay FEC. An alternative method would be for the exclusive, royalty-free license period to be only for a limited time, for example three years. At the end of this period, the university could grant a royalty free extension if the company has an ongoing development program. Otherwise, the university could require the company to pay higher royalties or make the technology available for licensing to other companies. This is essentially the recently adopted policy of the University of New South Wales, Australia, under its Easy Access IP initiative [Note S22]. If the university wants to share in some of the financial benefits from a windfall invention, perhaps it could include a homerun clause, as have Pennsylvania State University and the University of Minnesota.

Probably the vast majority of industry-sponsored research projects that do not involve scientist-to-scientist collaboration (i.e., contract research where university scientist perform, on their own, research commissioned by a company) would fall into this category of focused sponsored research.

However, in the case of blue-sky projects where the research is more exploratory, the agenda more determined by university scientists, and the potential outcomes less clear or more broad and far reaching, companies that want exclusive rights to emerging inventions ought not to have automatic exclusive rights, but rather should have a first option to negotiate such rights after the inventions arise. Exclusive licenses generally should include incentives for the company to develop the inventions, such as annual renewal fees or specific development milestones. The respondent companies in our sample that engaged in blue-sky research with US universities did not complain about such requirements. Also the public has an interest in universities ensuring that there are appropriate development incentives for potentially promising discoveries arising from public research support. If the company wants assurance in advance that it will have exclusive control over any emerging IP, it should be required to pay the full economic cost of the research, as is now the case in the UK, and some Canadian universities.

In the case of Japan, dropping the opt out clause from the standard University of Tokyo Joint Research Contract (in *italics* near the end of the preceding subsection) and using this clause as a model for all Japanese universities in the case of blue-sky projects, would partially avoid the lock-up of a large proportion of university discoveries. It would make the IP provisions of the standard Japanese sponsored research contracts similar to that of the University of Toronto. It would permit universities to grant third companies non-exclusive licenses to collaborative inventions if the collaborating company is not developing them. In effect, this would give co-inventing companies an automatic royalty-free non-exclusive license, not the automatic, royalty-free, exclusive license rights they currently usually enjoy. However, other companies might still hesitate to invest in developing these discoveries if the collaborating company continues to hold non-exclusive rights (as threatened to be the case with TeraView before the sponsoring company finally agreed that all rights would be licensed exclusively to TeraView [Case S7]).

Probably a better way to address the lock up problem and still preserve the uniquely attractive IP incentives in Japan for companies to collaborate with universities would be for both parties to agree that the universities would hold all rights to emerging IP but that the sponsors would have royalty free exclusive rights for a limited time (for example three years). After this time, the universities could let the companies retain royalty free exclusive rights if they are concertedly developing the technologies. Otherwise the companies ought to pay substantial royalties (approximating FEC) to retain control, at least in the case of blue-sky inventions.

As a general rule, should sponsors of blue-sky research have at least an automatic royalty-free, non-transferable non-exclusive license? All the UK Lambert Model Agreements provide this right, even Level 1, and one of the US companies engaged in a blue-sky project specifically made a plea for such a “common sense” rule.

However, a divergent perspective was offered by the four companies that specifically cited the importance of startups in carrying forward development to the point where the technology is appealing to large companies [Note S23]. University technology transfer officials acknowledge that even non-exclusive rights to discoveries arising from broad scope collaborations can block entrepreneurial university scientists from forming startups. Perhaps, in the case of blue-sky projects, an alternative to an automatic, perpetual non-exclusive rights is a an automatic non-exclusive license limited to two or three years, after which time negotiation with the university would be required to continue non-exclusive rights.

### 3 Suggestions Regarding Confidentiality and Publication Freedom

The situation with respect to publication rights is also complex. We know of no North American university whose official policies would permit a research sponsor to delay publications beyond twelve months. Any delays can be only to ensure that the sponsor's own confidential information is not revealed and to prepare patent applications. Furthermore, the publication of graduation theses can be delayed only for an extremely limited period of time, if at all. Nevertheless, half of the 26 North American companies that either were startups or described focused collaborations expressed concern about leakage of information to competitors. In a few of these cases, disputes about publication freedom nearly became deal breakers. In about four cases, there was a suggestion that the final compromise agreements gave the companies some rights to limit publication of information generated by university researchers. It is not clear if any of these companies ever actually insisted on changes to the manuscripts or whether any of the academic researchers felt their academic freedom was curtailed. Also most of these companies said the negotiation process in the face of push back from universities helped them to understand the importance of publication freedom. It is tempting to speculate that the near absence of disputes over publication rights in the UK interviews is due to the Lambert Agreements. In the other countries, it might be helpful to initiate a dialog between industry and academia to work out similar options or understandings with respect to publication rights.

The Japanese experience serves as a cautionary reminder of what might happen if companies are given the right to require changes to academic publications. Research sponsors frequently do review manuscripts and sometimes do insist that research findings not be disclosed. Moreover, self-censorship among academic researchers is common. It is not uncommon for junior faculty, post-doctoral researchers and occasionally some doctoral students to express unease about participating in industry funded projects *precisely because they expect limitations on their ability to publish results* [Note S24].

The practice of some companies treating the results of academic collaborations as trade secrets that are not to be published has been documented in the Japanese media, particularly in the case of collaborations related to industrial manufacturing processes. For example, a 2007 article in the Nikkei Financial Daily noted how the results of collaborative research at Tohoku University and other academic centers are often kept secret, largely out of concern about technology leakage to competing companies in countries such as China. The focus of the article was that not even patent applications are being filed, because the applications would be published after eighteen months and details about the technologies thus revealed. The gist of the article was not to criticize this practice, but rather to advocate an expansion of “prior user rights” under Japan's patent law [61]. Prior user rights permit companies that have made inventions (perhaps in collaboration with universities) to keep such discoveries secret while preserving their right to use the inventions in the event some other entity patents them in the future – in other words, to make the treatment of research discoveries as trade secrets a more attractive option.

Conversations with companies, government officials and even some academics, suggest such permitting collaborating companies to censor academic publications is generally not opposed in Japan. The situation is probably similar to that in South Korea [38]. Manufacturing technologies are harder to protect with patents than are products, and this is particularly so when the imitation is likely to occur abroad. Thus many Japanese manufacturers believe they must rely primarily on trade secrets to protect their production technologies in global markets, and this applies as well to manufacturing-related technologies arising from collaborations with universities.

However, the fundamental purposes of universities are education and the creation and dissemination of new knowledge. Collaborations that exclusively appropriate the commercial benefits of this knowledge but also prevent its dissemination are contrary to these fundamental purposes. This contradiction is particularly stark in the case of blue-sky research and when the sponsors are leveraging public research support. If companies strongly believe that publication of collaborative research findings will undermine their competitive business positions and they want the results treated as trade secrets, they are essentially asking universities to become publicly subsidized contract research laboratories. In such cases the rationale for public support for such research begins to fall away. And if the practice of locking up academic IP and restricting publications is widespread, the rationale for public support for academic research crumbles. If universities nevertheless permit sponsors to restrict the publication of new research findings, then they must alert researchers in advance and try ensure that non-participation in the project will not be detrimental for one's career. The UK practice of requiring the sponsor to pay full economic costs (FEC) is probably an appropriate model in such circumstances.

### 4 Summary Observations

Beyond these specific suggestions, the interviews show the complexity of the issues related to IP and publication freedom that frequently arise in the context of collaborative research. Companies, particularly startups and those engaged in targeted collaborations with universities, often need exclusive IP rights to discoveries arising from these projects. Yet if sponsors automatically exclusively control collaborative discoveries and face no development obligations, the lock-up problem becomes very real — especially in the case of broadly applicable inventions where the companies have paid only a fraction of the actual research costs. Similarly, companies sometimes are legitimately concerned that competitors will benefit if the results of sponsored research is disclosed. However, the basic societal value of academic research depends upon free flow of information. The interviews show that companies, and society as a whole, can benefit greatly from university collaborations, but that striking an appropriate balance between the interests of companies, universities, and society as a whole can be problematic. From these interviews emerges a plea for flexibility and judiciousness on the part of universities and companies in dealing with these complex issues. In a broader sense, universities have a responsibility of good stewardship over the discoveries emerging from collaborative research — for the benefit of all stakeholders. The ability to fulfill this responsibility cannot be built overnight. But there ought to be long-term commitments to develop these capabilities.

## Supporting Information

Case S1 Example of a research collaboration focused on instrument development that was structured as a consulting agreement.
(DOCX)

Case S2 Example of a collaboration in automated food inspection structured as an endowed university chair for research and training.
(DOCX)

Case S3 A collaboration that began as an effort to market data base software and evolved into collaboration with a spin-off developing a biomedical device.
(DOCX)

Case S4 A collaboration involving a grocery store chain focused on issues of supply chain management.
(DOCX)

Case S5 An interaction involving an aerospace company focused mainly on training of undergraduates in skills needed by the entire industry.
(DOCX)

Cases S6 Two manufacturers engaged in collaborations to address technical issues, but which also value the collaborations as opportunities to enhance training of future graduates with skills valuable to their industries.
(DOCX)

Case S7 How a startup pursuing development of a novel technology arose from collaboration between an electronics company and a university.
(DOCX)

Case S8 Spin-off founded by a faculty member with industry experience that became one of the pioneers in the field of plant produced pharmaceuticals.
(DOCX)

Case S9 A startup notes tensions in university collaborations related to publication freedom, and describes how a collaboration with a key scientist (whom it eventually hired) was structured as a consultation, so as to give the company greater control over publications.
(DOCX)

Case S10 An interaction that might be classified either as a typical or blue sky collaboration, involving consortium participation, facilities construction, and funding of visiting professorships and collaborative research.
(DOCX)

Cases S11 Two cases of collaborations involving small UK companies illustrating the limits of absorptive capacity in some small companies.
(DOCX)

Cases S12 Two examples of large, sophisticated manufacturers gaining new insights from collaborations with universities in the context of typical (relatively narrowly defined) collaborations. See also Case S3 and Case S10.
(DOCX)

Cases S13 Two examples of Canadian companies engaged in typical collaborations that wanted guarantees against information disclosure.
(DOCX)

Case S14 The only Japanese company that said that, as a matter of general principle, it supports freedom of collaborating university researchers to publish their findings and will not seek to restrict the content of academic publications.
(DOCX)

Cases S15 Two examples of successful KTP collaborations.
(DOCX)

Cases S16 Three UK companies engaged in “typical” collaborations describe overvaluation of IP and “incompetent” practices by technology management offices in prominent UK universities.
(DOCX)

Cases S17 Overview of a blue-sky consortium project in Japan that involved three interviewed companies, plus another Japanese and a Canadian blue sky project, noting the pressures the deep blue projects are facing to justify their continuation.
(DOCX)

Case S18 Overview of a deep blue collaboration involving a US chemical company, including IP terms often applicable to such collaborations.
(DOCX)

Case S19 A US pharmaceutical company engaged in deep blue collaborative initiatives where the company does not seek IP rights from the universities.
(DOCX)

Case S20 A UK company describing a deep blue collaboration, while noting problems caused by the university's overvaluing IP and objecting to having to pay FEC.
(DOCX)

Note S1 4-country comparison of university license vs industry sponsored research revenue.
(DOCX)

Note S2 Examples of how de novo research collaborations are initiated and how past personal connections are not always supportive of innovative collaborations.
(DOCX)

Note S3 Open Innovation Principles (Kauffman Foundation).
(DOCX)

Note S4 Reasons for excluding five organizations from the analysis, even though they engaged in relationships with universities akin to research collaborations.
(DOCX)

Note S5 More on “engineering – ICT” categorization.
(DOCX)

Note S6 More on “engineering – gov’t” categorization.
(DOCX)

Note S7 More on split classifications between technical fields.
(DOCX)

Note S8 Contact time with university collaborators, startups compared with large companies.
(DOCX)

Note S9 Additional information on the KTP program.
(DOCX)

Note S10 Examples of Canadian universities engaged in blue-sky collaborations expecting to own resulting inventions.
(DOCX)

Note S11 Praise for assays developed by a research consortium under which a company engaged in a blue-sky collaboration, even though the company suggested restructuring collaborations as consultancies in order to avoid paying FEC.
(DOCX)

Note S12 Evidence that American companies acceptance of US university IP management practices is probably not due to lack of patentable discoveries emerging from such research.
(DOCX)

Note S13 A major UK company (not among the interviewees for this study) indicating its acceptance of American university technology management practices.
(DOCX)

Note S14 The contractual basis for sponsoring companies to censor academic publications, taking as an example, the University of Tokyo's standard collaborative research contract.
(DOCX)

Note S15 Calculation and interpretation of industry co-ownership rates of Japanese university patents.
(DOCX)

Note S16 Costs covered (and not covered) by industry funding for sponsored research in Japanese universities, and international comparisons of industry contributions to total university R&D expenditures.
(DOCX)

Note S17 Abandonment (dedication to public) of about half of joint industry-university Japanese patent applications.
(DOCX)

Note S18 Lambert Agreement URLs.
(DOCX)

Note S19 URLs of various Canadian universities regarding publication rights and ownership of IP arising under industry sponsored research.
(DOCX)

Note S20 The evolution of Japan's pre-2004 university technology transfer system.
(DOCX)

Note S21 URLs for University of Minnesota and Penn State University policies permitting industry.
(DOCX)

Note S22 URL describing University of New South Wales's Easy Access IP policy.
(DOCX)

Note S23 Large companies that explicitly stated that independent startups play a vital role in developing early stage discoveries to the point where they are attractive to large companies.
(DOCX)

Note 24 Source for assertion that Japanese junior faculty and some graduate students are hesitant to engage in industry sponsored projects out of concern that they cannot publish their findings.
(DOCX)

Table S1 All companies and universities that were subjects of the case studies (listed alphabetically and unmatched).
(DOCX)
