# Supplementary material for: Industry-University Collaborations in Canada, Japan, the UK and USA – With Emphasis on Publication Freedom and Managing the Intellectual Property Lock-Up Problem
Source: PLoS One. 2014 Mar 14;9(3):e90302. doi: 10.1371/journal.pone.0090302 (PMC3954545; doi:10.1371/journal.pone.0090302)
Supplement: Case S1 — Example of a research collaboration focused on instrument development that was structured as a consulting agreement. (DOCX) [file pone.0090302.s001.docx]

Case S1:

This company specialized in machine tools but was seeking to expand into an area less subject to business cycle fluctuations. A university professor with expertise in Ramen spectroscopy was known to the scientist heading the company’s laser interferometry unit. (Their acquaintance dated from the professor’s consultation with the company scientist’s previous employer.) The relationship began informally with a key role being played by one of the professor’s doctoral students whose dissertation research on increasing the detection speed of charge coupled detectors was relevant to developing commercial Ramen spectrometers. When the company decided it was going to make an effort to develop and market such spectrometers, the student joined the company and the professor became a formal consultant helping to refine the system and to develop a second, more advanced, prototype.
