# Supplementary material for: Industry-University Collaborations in Canada, Japan, the UK and USA – With Emphasis on Publication Freedom and Managing the Intellectual Property Lock-Up Problem
Source: PLoS One. 2014 Mar 14;9(3):e90302. doi: 10.1371/journal.pone.0090302 (PMC3954545; doi:10.1371/journal.pone.0090302)
Supplement: Case S2 — Example of a collaboration in automated food inspection structured as an endowed university chair for research and training. (DOCX) [file pone.0090302.s002.docx]

Case S2:

The company, a subsidiary of a German food processing machinery company, specialized in machine vision systems for inspecting fish prior to processing. It had collaborated previously with this university in Canada’s Maritime Provinces and the professor who was to hold the chair was well known to the company. The company’s main goal in establishing the chair was to train students in the machine vision field and to have more graduates with relevant skills whom it could hire. It also wanted to ensure that research would be conducted in areas important for its business. It felt that, heretofore, too much engineering research in the university was devoted to oil and gas and not enough the fishing industry, the traditional industry of this province. A large matching increment of national and provincial government co-funding helped convince the German parent to make the investment to establish the chair.

About a year after the interview, the company went out of business. It is not clear whether the activities under this chair produced any significant innovations or patent filings prior to dissolution.
