# Supplementary material for: Industry-University Collaborations in Canada, Japan, the UK and USA – With Emphasis on Publication Freedom and Managing the Intellectual Property Lock-Up Problem
Source: PLoS One. 2014 Mar 14;9(3):e90302. doi: 10.1371/journal.pone.0090302 (PMC3954545; doi:10.1371/journal.pone.0090302)
Supplement: Case S3 — A collaboration that began as an effort to market data base software and evolved into collaboration with a spin-off developing a biomedical device. (DOCX) [file pone.0090302.s003.docx]

Case S3:

The respondent was a large database software company that had a history of developing software applications for the UK’s National Health Care System and care providers. It felt that some of its systems related to medical messaging might be applicable to monitoring of individual patients. The company had longstanding contacts with a major university medical center, which it contacted about co-development of patient applications. The university referred the company to its technology commercialization office. There was perception of a “lack of connectivity” between this office and the relevant university biomedical research laboratories. When contact was finally made, discussions focused on applying the company’s software to an experimental device that could transmit in real time a patient’s vital sign information to a remote monitoring station, while also linking to the patient’s medical record. The specific project involved the company funding two doctoral students to develop algorithms to determine which real-time data patterns warranted the system sounding an alert, and also preparing for clinical trials. The relationship with the doctoral students did not develop as hoped. Data were lost. Results were not written up. Also the company would have liked to interact with clinicians and patients to obtain their feedback, but this did not happen for a variety of reasons, including lack of senior staff in the company available for the project.

However, key university scientists formed a startup to commercialize the remote monitoring technology. Thus a parallel effort began, which soon became the driving development force. The company interacted closely with this startup. However, it concluded its collaboration with the university after 30 rather than the planned 36 months, in part because the startup was moving forward on its own into clinical trials. At the time of the interview, the device had not been approved by the US Food and Drug Administration (FDA). Thus, the company thought the startup’s revenue prospects were limited.

Despite the interaction with the university not having been perfectly smooth, the company thought it had gained and contributed much. The startup had identified a number of issues key to make remote health monitoring devices and software work in real life situations. The university was also good at recognizing these “real problems” and coming up with ideas how to deal with them. The company learned a lot from listening to the university and “hearing them think out loud” – not only about technical issues but also about the university’s own shifting objectives. Through the university, the company made connections that would not otherwise have made – e.g., with investors and pharmaceutical companies – and it shared these contacts with the startup. The company learned about FDA compliance procedures. It has moved on to work other startups in the field of ubiquitous health-related computing, at least one of which had an FDA approved product at the time of the interview.

In 2011, three years after the interview, the startup featured in this report obtained FDA approval for its device using software developed from its interaction with the company.
