# Supplementary material for: Industry-University Collaborations in Canada, Japan, the UK and USA – With Emphasis on Publication Freedom and Managing the Intellectual Property Lock-Up Problem
Source: PLoS One. 2014 Mar 14;9(3):e90302. doi: 10.1371/journal.pone.0090302 (PMC3954545; doi:10.1371/journal.pone.0090302)
Supplement: Case S4 — A collaboration involving a grocery store chain focused on issues of supply chain management. (DOCX) [file pone.0090302.s004.docx]

Case S4:

The director of business partnerships in a center for environmental research and teaching in a UK university contacted regional food retailers about possible collaborations. The company, a chain of grocery stores emphasizing quality produce, fair trade, and working directly with farmers and suppliers, responded. The company saw an opportunity to investigate specific areas of concerns, such as agronomy, environment, processing and packaging. Projects were selected in consultation between the university, the company and its supply chain members. Some projects were outsourced to outside organizations. Some involved literature reviews that could be done by university researchers. Short-term duration (e.g. one week) training courses primarily for supply chain members also became an important activity. Overall, one of the main benefits of the interaction was the establishment of direct communication between suppliers and university personnel.
