# Supplementary material for: Industry-University Collaborations in Canada, Japan, the UK and USA – With Emphasis on Publication Freedom and Managing the Intellectual Property Lock-Up Problem
Source: PLoS One. 2014 Mar 14;9(3):e90302. doi: 10.1371/journal.pone.0090302 (PMC3954545; doi:10.1371/journal.pone.0090302)
Supplement: Case S5 — An interaction involving an aerospace company focused mainly on training of undergraduates in skills needed by the entire industry. (DOCX) [file pone.0090302.s005.docx]

Case S5:

A manufacturer of jet engines was concerned that engineering programs in North American universities were not training undergraduates in the skills the company needed. Along with other aerospace companies, this company established a training and research institute in 2001 within a university near to its main Canadian facilities. The institute describes it main mission as industry-driven, project-based learning for undergraduates. The company also sponsors research projects in the institute in coordination with an aerospace consortium project supported by the provincial government. However the company considers human skills development to be more beneficial compared to research findings. This institute has helped alleviate its need, and the needs of other aerospace companies, for skilled graduates whom they can hire. Despite this emphasis on education, the company insisted that all students and faculty involved in its learning projects sign non-disclosure agreements and that the company own any patentable inventions that might emerge.
