# Supplementary material for: Industry-University Collaborations in Canada, Japan, the UK and USA – With Emphasis on Publication Freedom and Managing the Intellectual Property Lock-Up Problem
Source: PLoS One. 2014 Mar 14;9(3):e90302. doi: 10.1371/journal.pone.0090302 (PMC3954545; doi:10.1371/journal.pone.0090302)
Supplement: Cases S6 — Two manufacturers engaged in collaborations to address technical issues, but which also value the collaborations as opportunities to enhance training of future graduates with skills valuable to their industries. (DOCX) [file pone.0090302.s006.docx]

Cases S6:

Example 1: A major manufacturer of heavy construction equipment described collaborations with four Japanese universities addressing a variety of issues related to machine power efficiency and pollution reduction. The company perceived that access to university expertise in these areas would help its development operations. However, it also perceived that it needed young engineering graduates with high value skills in areas such as metallurgy. Thus the research collaborations provided the secondary benefit of increasing the pool or potential recruits with these skills. The company took the then unusual step of offering stipends to some graduate students involved in the collaborative projects. It also is involved in government-coordinated programs to train students in fields of critical importance to Japanese industry. Some of the programs involve the company accepting university students as interns – still a relatively uncommon practice among Japanese manufacturing companies.

Example 2: This case involves a major manufacturer of specialty steel and steel products. The interview focused mainly on collaborations with a regional university related to manufacturing processes and also to utilizing unique metallurgical analytical instruments and expertise in the university. However, the company also spoke favorably about participation in the Advanced Research and Education Center for Steel (ARECS) located in the engineering department of a well known, but distant, national university. ARECS activities are supported by the company and about twelve other Japanese steel manufacturers. The company considers the main benefit of ARECS to be training in fields of metallurgy crucial to the company and to the steel industry in general.
