# Supplementary material for: Industry-University Collaborations in Canada, Japan, the UK and USA – With Emphasis on Publication Freedom and Managing the Intellectual Property Lock-Up Problem
Source: PLoS One. 2014 Mar 14;9(3):e90302. doi: 10.1371/journal.pone.0090302 (PMC3954545; doi:10.1371/journal.pone.0090302)
Supplement: Case S7 — How a startup pursuing development of a novel technology arose from collaboration between an electronics company and a university. (DOCX) [file pone.0090302.s007.docx]

Case S7:

This startup arose from collaborative research between the European branch of a Japanese electronics company and Cambridge Cavendish Laboratory into terahertz frequency light, i.e., light whose spectrum falls between that of infrared light and radio waves. The research included instrument development and potential practical applications. The electronics company decided not to commercialize this technology. However, several of the scientists were enthusiastic about starting a company to commercialize applications in fields such as microscopy, materials testing, medical imaging, detection of hazardous substances, and quality control in pharmaceutical and semiconductor manufacturing. These scientists had lined up venture capital investment, and one was prepared to leave his position with the collaborative project to lead the company. Under the collaboration with Cambridge, the Japanese company had exclusive rights over the emerging patents -- patents that would cover the startup’s core business. The potential investors refused to invest unless the company secured exclusive licenses of this IP. The European research branch of the electronics company supported transfer of exclusive rights to the startup. However, the home office hesitated because of a longstanding corporate policy against exclusively out-license technology (a policy common in the Japanese electronic industry) and also because Japanese electronics companies felt obligated to make available to each other, on a non-exclusive basis, IP obtained through overseas academic collaborations. After much back and forth, the home office finally agreed to make an exception and to license the patents exclusively to the startup. The deciding considerations were, first, that the technology would probably have died if the startup were not formed and there would have been no benefit to the parent, and second, the enthusiasm and level-headed commitment of the scientists towards starting a company.
