# Supplementary material for: Industry-University Collaborations in Canada, Japan, the UK and USA – With Emphasis on Publication Freedom and Managing the Intellectual Property Lock-Up Problem
Source: PLoS One. 2014 Mar 14;9(3):e90302. doi: 10.1371/journal.pone.0090302 (PMC3954545; doi:10.1371/journal.pone.0090302)
Supplement: Case S8 — Spin-off founded by a faculty member with industry experience that became one of the pioneers in the field of plant produced pharmaceuticals. (DOCX) [file pone.0090302.s008.docx]

Case S8:

The drugs-from-plants spin-off was founded by a professor who had spent his early career in industry where he pioneered the development of the first transgenic oilseeds. At the university, he developed a platform technology involving genetic engineering of safflower plants so that their seeds would contain therapeutic proteins such as insulin. The negotiations to form the spin-off took place in an environment of uncertainty as to how the business would evolve, and the original license was put together quickly by persons without much experience. In hindsight, the terms of the original license were insufficiently flexible on certain issues, particularly the scope of technologies included in the license. The license was essential to establishing the company and its receiving private investment. The spin-off subsequently received investments from not only venture capital but also a major agricultural chemical and seed company. In 2011, it was among three companies worldwide that were farthest along in human trials to develop plant-manufactured therapeutics for human use.* However, the next year it filed for bankruptcy, probably because it could not meet mounting costs associated with clinical trials and with purifying its plant-produced insulin.

* Thomas DR, Penney CA, Majumder A, Walmsley AM (2011) Evolution of plant-based pharmaceuticals. Int’l J Molecular Sciences 12: 3220-3236.
