# Supplementary material for: Industry-University Collaborations in Canada, Japan, the UK and USA – With Emphasis on Publication Freedom and Managing the Intellectual Property Lock-Up Problem
Source: PLoS One. 2014 Mar 14;9(3):e90302. doi: 10.1371/journal.pone.0090302 (PMC3954545; doi:10.1371/journal.pone.0090302)
Supplement: Case S9 — A startup notes tensions in university collaborations related to publication freedom, and describes how a collaboration with a key scientist (whom it eventually hired) was structured as a consultation, so as to give the company greater control over publications. (DOCX) [file pone.0090302.s009.docx]

Case S9:

The company, which designs life support systems for use in extreme environments, began collaborating with a neighboring university when an adjunct faculty member approached the company about the possibility of working in the company while maintaining her university laboratory. The company recognized the value of the professor’s expertise in oxygen generating fuel cells. However, negotiations with the university to fund research in her laboratory were difficult. The company saw the research as part of a consultation, while the university interpreted it as creation of new ideas – and thus wanted to ensure publication rights. The parties worked out an agreement under which the relationship was recognized primarily as a consultation, but the university researcher retained the right to publish while the company had the right of pre-publication review. The low likelihood that any publications would arise from this work was a factor in the company agreeing to this compromise.

The faculty member was eventually hired by the company and her knowledge brought in house. She helped the company to win contracts. Her scientific publications as a company scientist raised the profile of the company. Engagement with the university broadened to include student internships, teaming up with the university on government sponsored projects, and additional consultations with university researchers. These consultations helped to verify the company’s designs and speed up the product development cycle. The company was very pleased with how the relationship with the university developed.

Nevertheless, the company remains reluctant to conduct large-scale research in universities “because of tensions in the relationship that can arise when highly valuable, proprietary know-how enters the relationship.” It prefers to use university know-how and expertise in an advisory role in order to avoid complicating issues such as determining ownership of intellectual property, handling of proprietary information, and conflicting opinions on publication rights. However, the company respondent concluded that these problems can be mitigated by what he called the Golden Rule: If the company covers all the costs of a research project on its own, these issues can (or ought to be) easily resolved.
