# Supplementary material for: Industry-University Collaborations in Canada, Japan, the UK and USA – With Emphasis on Publication Freedom and Managing the Intellectual Property Lock-Up Problem
Source: PLoS One. 2014 Mar 14;9(3):e90302. doi: 10.1371/journal.pone.0090302 (PMC3954545; doi:10.1371/journal.pone.0090302)
Supplement: Case S10 — An interaction that might be classified either as a typical or blue sky collaboration, involving consortium participation, facilities construction, and funding of visiting professorships and collaborative research. (DOCX) [file pone.0090302.s010.docx]

Case S10

This Japanese manufacturer of machines for quality control testing of computer chips during various stages during the chip manufacturing process joined a VLSI design and education consortium based in a major Japanese university. Other members included chip manufacturers and other universities. The company felt it should join in order to anticipate advances in chip design and manufacturing and to be able to design testing equipment for future manufacturing processes. It also wanted to encourage more skilled graduates to enter the chip design and testing industries. It joined in a big way: donating a building and also funding two visiting professorships, one filled by and American and the other by a German academic, along with a supporting assistant professorship. In addition, the company funded at least three joint research projects, all with the visiting professors.

It said it expected to co-own inventions made under these projects, but mainly to ensure freedom to operate. It added that, in case of collaborations with US universities, it would be satisfied in most cases with non-exclusive licenses. This mechanism of collaborating with universities by funding research professorships is common among Japanese companies.

As noted in the text, there is a question whether this project ought to be classified as blue-sky. The decision to classify it a typical collaboration was based upon the supposition that the joint research projects it funded were relatively limited in scope with research focuses primarily determined by the company. If this is not the case, the project probably should be classified as blue-sky. Among all the cases, this was the most problematic in terms of classification.
