# Supplementary material for: Industry-University Collaborations in Canada, Japan, the UK and USA – With Emphasis on Publication Freedom and Managing the Intellectual Property Lock-Up Problem
Source: PLoS One. 2014 Mar 14;9(3):e90302. doi: 10.1371/journal.pone.0090302 (PMC3954545; doi:10.1371/journal.pone.0090302)
Supplement: Cases S11 — Two cases of collaborations involving small UK companies illustrating the limits of absorptive capacity in some small companies. (DOCX) [file pone.0090302.s011.docx]

Cases S11

Example 1: A company with only 23 employees whose business is manufacturing of specialty fabrics and textile finishings described a number of interactions with universities over the previous ten years. However, these tended to be initiated through random personal connections, to be focused on specific one-off problems (e.g., how to apply a polymer to cotton to create artificial chamois leather), to be structured as informal consultations, and “to live only as long as the project lasts.” The company expressed aversion towards university or government outreach programs that involve “administrative weight and bureaucracy” and said that it simply could not meet the salary expectations of most university graduates. It did suggest that some systematization of matching small company needs with university resources would be helpful – and that trade associations might play a role in this regard.

Example 2: A company making glue guns and adhesives for international markets applied through the KTP program (described below) for a recent university graduate (a.k.a., Associate) who could help it develop an e-commerce system. The Associate was employed for two years with faculty supervision. With considerable input from one of the company employees, an e-commerce system was developed which has positively affected sales. The company described the working relationship as very good. However, project meetings consumed a lot of scarce staff time. “The need to match the graduate with scarce personnel resources in the company capable of mentoring, embedding, and creating a productive interaction was a ‘strain’.” However, at the end of the KTP period, the Associate continued to work with the company on a part-time basis and went on to gain a PhD, suggesting long term benefits and academic linkages from the interaction. The respondent noted that few of the company’s 35 employees have university degrees.
