# Supplementary material for: Industry-University Collaborations in Canada, Japan, the UK and USA – With Emphasis on Publication Freedom and Managing the Intellectual Property Lock-Up Problem
Source: PLoS One. 2014 Mar 14;9(3):e90302. doi: 10.1371/journal.pone.0090302 (PMC3954545; doi:10.1371/journal.pone.0090302)
Supplement: Cases S12 — Two examples of large, sophisticated manufacturers gaining new insights from collaborations with universities in the context of typical (relatively narrowly defined) collaborations. See also Case S3 and Case S10. (DOCX) [file pone.0090302.s012.docx]

Cases S12

Example 1: A professor in a major UK university who had already founded a medical imaging startup approached the person in charge of software development for the UK branch of a multinational IT company. Knowing that the company was interested in providing equipment and services to the UK National Health System’s basic IT infrastructure, the professor proposed a joint application for government research funding related to establishing a nationwide database of breast cancer related radiographic images to aid in the diagnosis and planning of therapy for individual patients. The joint application was successful, and company and university researchers worked closely together. Resistance in the medical community to using remote radiologists ultimately prevented the project from being implemented. Nevertheless, the company felt that working with the university helped to conceptualize problems in a more global and theoretical fashion resulting in more robust and mathematically sound software. Major problems were solved related to developing new approaches to running a large database over multiple institutions, particularly with respect to grid computing and software content management. No university IP emerged.

Example 2: A jet engine manufacturer approached professors in a well-known aeronautical engineering department about ways to cool sound abatement systems in an environment subject to high-pressure waves. The collaboration produced results contrary to expectations. The university proposed interesting solutions, which the company implemented in a crash program. In the process, a new discipline, unsteady fluid dynamics, was created. Insights from this collaboration helped to understand how unsteady pressures affect other jet engine components. This became the focus of subsequent university collaborations. The company noted that the number of times its researchers initiate meetings on their own with university researchers is a useful metric of the value of a interaction, and judging from this criterion, the collaboration was very successful. The company values patents that emerge from these collaborations. Generally it wants to be assured it will be free to use the patented discoveries.
