# Supplementary material for: Industry-University Collaborations in Canada, Japan, the UK and USA – With Emphasis on Publication Freedom and Managing the Intellectual Property Lock-Up Problem
Source: PLoS One. 2014 Mar 14;9(3):e90302. doi: 10.1371/journal.pone.0090302 (PMC3954545; doi:10.1371/journal.pone.0090302)
Supplement: Cases S13 — Two examples of Canadian companies engaged in typical collaborations that wanted guarantees against information disclosure. (DOCX) [file pone.0090302.s013.docx]

Cases S13

Example 1: The statement was made by a company that has had a longstanding relationship with an academic laboratory assisting in the development of collision avoidance software for unmanned surveillance aircraft. The company believes these collaborations have been smooth and fruitful. Both the dean and main professor have industry experience and are attuned to industry needs. Patents rarely emerge because most of the technologies are known. Projects depend on integrating these technologies in a useful fashion in the context of unmanned aerial vehicles. It was not clear from the transcripts whether NDAs are important merely in order to prevent disclosure of pre-existing confidential company information, or because they also extend to information generated by university researchers. National security as well as competitive business concerns may have been involved in this case.

Example 2: This case involved a company that manufactures vehicle steel frames. It said that some of the collaboration data related to safety testing should not be disclosed for competitors to see. Such a request that data for regulatory agencies not be made available to competitors would probably be considered reasonable in most situations.
