# Supplementary material for: Industry-University Collaborations in Canada, Japan, the UK and USA – With Emphasis on Publication Freedom and Managing the Intellectual Property Lock-Up Problem
Source: PLoS One. 2014 Mar 14;9(3):e90302. doi: 10.1371/journal.pone.0090302 (PMC3954545; doi:10.1371/journal.pone.0090302)
Supplement: Case S14 — The only Japanese company that said that, as a matter of general principle, it supports freedom of collaborating university researchers to publish their findings and will not seek to restrict the content of academic publications. (DOCX) [file pone.0090302.s014.docx]

Case S14

This company often engages universities to assist in evaluating new communications technologies, particularly software algorithms. The case in point involved collaboration with a major university on evaluating a coding system that facilitates correction of errors that inevitably arise in long distance wireless transmissions. The collaboration was initiated by contacts between company and university researchers made at academic conferences. The project continued for two or three years and focused on development of tools to measure the coding system’s performance and possible applications. University and company researchers worked closely together, and the former developed helpful interpretive algorithms. As is often the case with this company’s university collaborations, patentable inventions did not arise; however, patents did arise from in-house basic and development teams working together and building upon the university collaboration. However, when patentable discoveries do arise in the context of university collaborations, the company expects to co-own the patents. Uniquely among all the interviewed Japanese companies, the respondent said that his company does not try to control the content of academic publications (aside from making sure the company’s own trade secrets are not revealed) and it supports the freedom of academic researchers to publish their findings.
