# Supplementary material for: Industry-University Collaborations in Canada, Japan, the UK and USA – With Emphasis on Publication Freedom and Managing the Intellectual Property Lock-Up Problem
Source: PLoS One. 2014 Mar 14;9(3):e90302. doi: 10.1371/journal.pone.0090302 (PMC3954545; doi:10.1371/journal.pone.0090302)
Supplement: Cases S15 — Two examples of successful KTP collaborations. (DOCX) [file pone.0090302.s015.docx]

Cases S15

Case 1: The KTP program helped turn around a Scottish manufacturer of windows and doors. The company was losing money and realized it had to be more “engineering and technology driven.” The KTP office helped identify an academic in a nearby university who was interested in working with the 200-person company. Two graduates agreed to be Associates. At first the company thought the emphasis ought to be on “lean” manufacturing. However, the Associates and the academic supervisor determined that the company needed to reorient its focus to being more attuned to customer preferences and quickly adapting its product offerings and manufacturing to meet those preferences. The company established in-house design capabilities. The entire shop floor became involved with the Associates who were embedded as part of the company team. The company board gave high priority to the KTP project. The Associates stayed on, one as head of the machine shop, the other as head of design. Between 1997 and 2007, the company turned from loss to profit and production increased over threefold.

Case 2: In the case of a water utility serving northwest England, KTP projects have become a centerpiece of the utility’s entire R&D effort. At the same time, they have facilitated decentralization of R&D and sharing of research findings across the entire organization. Before becoming involved in KTP projects, most of the utility’s R&D was either centralized or outsourced through an industry-wide research consortium. Collaborations with universities relied on personal contacts and had to be negotiated from scratch each time. Engaging universities through the KTP program standardized the negotiation and implementation process. Having a new university graduate work for two years at a highly subsidized salary with university mentorship was more attractive than funding research in university laboratories. The program helped to disperse the utility’s R&D efforts to the plants/regions were problems had been identified or where university expertise was strong. Thus phosphate removal was pursued in Liverpool, network simulation in Sheffield, and carbon flux, enzyme hydrolysis, microbial management, and agricultural waste cake in other locations. Software developed through KTP partnerships has increased efficiency of wastewater treatment. The program has stimulated tracking and communication of research benefits within the company. The utility’s R&D efforts are now more networked with outside centers of expertise. Alone among all the six KTP cases, this company said that it often wants to be assured that it will co-own any inventions that arise from university collaborations and that it usually requires a separate side agreement addressing IP to accompany the standard KTP agreement.
