# Supplementary material for: Industry-University Collaborations in Canada, Japan, the UK and USA – With Emphasis on Publication Freedom and Managing the Intellectual Property Lock-Up Problem
Source: PLoS One. 2014 Mar 14;9(3):e90302. doi: 10.1371/journal.pone.0090302 (PMC3954545; doi:10.1371/journal.pone.0090302)
Supplement: Cases S16 — Three UK companies engaged in “typical” collaborations describe overvaluation of IP and “incompetent” practices by technology management offices in prominent UK universities. (DOCX) [file pone.0090302.s016.docx]

Cases S16

Three companies voiced this complaint, each time in relation to a prominent UK university.

Case 1: This involved a collaboration built around two doctoral students working on two company-sponsored computer gaming and animation thesis projects. Although the company’s primary goal was recruitment and public relations, and one of the students left early without completing the thesis, the company felt the students interacted well with company researchers and there were many diffuse “knowledge and cultural” benefits for the company. Moreover, the single completed thesis presented knowledge that would likely be incorporated in a forthcoming software product. However, university lawyers and technology managers nearly derailed the project according to the company. They overvalued the university IP and underestimated the effort the company would have to invest to realize commercial value from it. They continually argued about ownership and royalties. In the end, the company decided to publish the source code that was at the heart of student’s thesis – thereby dedicating it to the public and ending disputes with the university over royalties and ownership.

Case 2: This case was initially presented under Case S1. As a result of the consultancy, the main patent was assigned to the company. However, the university patented some additional functionality. The company considered the improvement marginal, but nevertheless sought a license. A long negotiation ensued. The company attributes this to lack of business awareness and valuation expertise on the part of university technology transfer staff, which lead them to overvalue the IP and to overlook the threat of royalty stacking which might make the final product uncompetitive. The company also was put off by university’s trying to condition a license on the company’s agreeing to provide more research funding. The company indicated it had considered forming spin-offs based on university research, but felt that university IP management staff did not have enough expertise to secure the sufficiently detailed and comprehensive patent protection that would be necessary to form such spin-offs.

Case 3: The company described several civil engineering collaborative projects with universities in America, Asia, Australia and the UK. It mentioned aggressive IP management and the ensuing legal debates as two factors slowing progress in the collaborations, and singled out the practices of a major UK university.
