# Supplementary material for: Industry-University Collaborations in Canada, Japan, the UK and USA – With Emphasis on Publication Freedom and Managing the Intellectual Property Lock-Up Problem
Source: PLoS One. 2014 Mar 14;9(3):e90302. doi: 10.1371/journal.pone.0090302 (PMC3954545; doi:10.1371/journal.pone.0090302)
Supplement: Cases S17 — Overview of a blue-sky consortium project in Japan that involved three interviewed companies, plus another Japanese and a Canadian blue sky project, noting the pressures the deep blue projects are facing to justify their continuation. (DOCX) [file pone.0090302.s017.docx]

Cases S17

Example 1:

Three of the eight Japanese companies described blue-sky collaborations that were part of a multi-company consortium collaboration with a single Japanese university. This research consortium deals with electro-organic-chemical devices, e.g., organic electroluminescence displays and polymer solar cells. Two of these companies had specific applications in mind, one related to solar cells, the other to devices and flexible displays. But for both, realization of a marketable product might be years away. These were classified as light blue collaborations. However, the third company’s overall focus was closely aligned with that of the university laboratory. Its role was to work with the university researchers to develop new chemicals that the other companies in the consortium could develop and incorporate in future products. In fact, the entire consortium project was initiated through discussions between the Chief Technology Officer of this company and a well-known chemist heading the academic laboratory. Thus, this third company’s participation in the consortium was classified as a deep blue collaboration.

None of the three companies pointed to specific benefits, except that the deep blue participant mentioned development of analytical and modeling software. However, all three companies praised the collaboration for opening up communication between them and the university, and among the five companies involved in the consortium. “It exposes our researchers to a lot of new ideas,” remarked the Director for Research of one of the companies. Interestingly, the three companies also commended the project for building networks among the university researchers themselves. “Device and materials professors didn’t communicate with each other before this project,” said the above-quoted Research Director.

However, the deep blue respondent was hesitant about the value of the project. He noted that there were no plans to launch more large-scale collaborations with universities until the results of this project were clearer. He also remarked that this project could not be continued for many more years without showing specific benefits.

Example 2:

A telecommunications company sponsoring IT-related research in several Canadian universities (one of the two deep blue collaborations described by Canadian companies) made a similar remark. “The actual impact that this project has had on the company’s value chain is difficult to ascertain. There have not been a lot of research results that can be traced directly into products.” It noted that ensuring access to cutting edge university research has been valuable. Also, the project “fulfills some aspects of the company’s corporate social responsibility mandate.” However, with senior corporate management changes on the horizon, the respondent expressed concern about how new management might deal with corporate social responsibility issues in the future.

Indeed, it seems as if the project has not been active since about 2009.

Example 3:

The other deep blue collaboration described in the Japanese interviews (besides the one mentioned in Example 1, above) involved a pharmaceutical company collaborating with a well-known university to promote drug discovery across a wide therapeutic area. Half of the multi-million dollar annual project costs are co-funded by the Japanese Government. Mid-term evaluations by the funding agency focused, in part, on tangible benefits. This company, and others participating in other research projects within the same government co-funded program, felt that in order not to lose government funding, they had to show tangible achievements, such as analytical devices, even though such devices they may not represent the fundamental goals of the project. Nevertheless, some drug candidates arising under the collaboration are moving forward towards clinical trials.
