# Supplementary material for: Industry-University Collaborations in Canada, Japan, the UK and USA – With Emphasis on Publication Freedom and Managing the Intellectual Property Lock-Up Problem
Source: PLoS One. 2014 Mar 14;9(3):e90302. doi: 10.1371/journal.pone.0090302 (PMC3954545; doi:10.1371/journal.pone.0090302)
Supplement: Case S18 — Overview of a deep blue collaboration involving a US chemical company, including IP terms often applicable to such collaborations. (DOCX) [file pone.0090302.s018.docx]

Case S18

One of these is a chemical company that in 2000 began a wide-ranging initiative with a well-known university. The intention was to link the university’s fundamental scientific expertise in biochemistry with projects in the company (e.g., related to bio-polymers or bio-fuels) and also to generate ideas for new projects. In 2005, the project was renewed for an additional five years and the scope broadened to include chemical engineering, material science and nanotechnology. Sub-projects included both collaborative research and educational activities. Over the first seven years, nearly 50 sub-projects were funded involving 75 university faculty. Some of these sub-projects have lead to new products, but even those that have not have increased the company’s ability to solve problems. The company described this as by far the largest and most complex university collaboration it has ever undertaken.

Agreement on IP management was important for the collaboration, and it followed standard US university practices: If a company researcher works closely with a university researcher and makes an invention, it will be jointly owned by the university and company. If the only inventors are university researchers, the university retains ownership. In this latter case, the company will have an automatic non-exclusive license to certain discoveries (probably those within its business scope that it plans to use), and for such inventions it also has the first right to negotiate an exclusive license. The respondent, a company Manager responsible for this project, expressed no reservations about these terms.
