# Supplementary material for: Industry-University Collaborations in Canada, Japan, the UK and USA – With Emphasis on Publication Freedom and Managing the Intellectual Property Lock-Up Problem
Source: PLoS One. 2014 Mar 14;9(3):e90302. doi: 10.1371/journal.pone.0090302 (PMC3954545; doi:10.1371/journal.pone.0090302)
Supplement: Case S19 — A US pharmaceutical company engaged in deep blue collaborative initiatives where the company does not seek IP rights from the universities. (DOCX) [file pone.0090302.s019.docx]

Case S19

The pharmaceutical company described two funding programs to support university research. The first program provides grants, usually for three years, to support research that will help the company identify and nurture emerging technologies related to health care and to assess their risks and business potential. Often the company will announce particular topics of interest and invite research proposals in those areas. Although the projects usually do not involve company and university scientists working together, each project has a company liaison person who also serves an advisory role.

A more recent program involves the company and universities each contributing equally to a seed fund to support relatively small-scale interdisciplinary translational research projects to help move a technology closer to proof of concept. In these sub-projects, which typically last a year, company and university researchers often work together. At the time of the interviews, the company was collaborating with about twelve universities under this program.

Both of these programs are still ongoing. The respondent, the director of the corporate office that manages these programs, said that the company is experiencing a wide array of impacts, including increased staff knowledge, direct contributions to product development, product improvements and creation of new products. Drug eluting stents was cited as a specific example of a product to emerge from these programs.

The respondent stressed the company does not seek IP rights from these projects. In other words, the universities own the research results and are under no obligation to grant the company preferential licensing rights. “[The company] wants to gain new knowledge from these programs, not necessarily new IP.”

The respondent noted that the parties’ initial perceptions regarding the value of collaborative inventions often differ greatly. The university usually considers the value of a final product and thus values its discoveries highly, while the company considers the long development process and the risks of failure and thus values the discoveries much less. If technology transfer negotiations are conducted at the time IP arises (at the time inventions are made), these differing perceptions often result in disagreements. At least in pharmaceuticals, it makes sense to postpone these discussions until more research has been done and the discoveries are closer to proof of concept (e.g., human trials). At this point, value perceptions are closer and negotiations are easier.

The company does expect to be able to work on collaborative discoveries in its in-house laboratories. It hopes that by not laying claim to IP, it will generate good will and facilitate ongoing communication with the university. As both sides advance the science, if the university seeks a development partner, the company hopes to be first in line for partnership discussions. From the company’s perspective, it is fine if the university forms a startup and obtains venture capital to advance a drug candidate or device towards clinical trials. If, as is often the case in the clinical trial stage, the startup seeks a licensee or a company that will buy it out, the company sees itself as being a likely partner, and at this point, disagreements about valuation are likely to be relatively easy to resolve.

Additional observations by the respondent are in the text under FINDINGS PERTAINING TO COLLABORATIVE RESEARCH, §4.2.
