# Supplementary material for: Industry-University Collaborations in Canada, Japan, the UK and USA – With Emphasis on Publication Freedom and Managing the Intellectual Property Lock-Up Problem
Source: PLoS One. 2014 Mar 14;9(3):e90302. doi: 10.1371/journal.pone.0090302 (PMC3954545; doi:10.1371/journal.pone.0090302)
Supplement: Case S20 — A UK company describing a deep blue collaboration, while noting problems caused by the university's overvaluing IP and objecting to having to pay FEC. (DOCX) [file pone.0090302.s020.docx]

Case S20

The company described a deep blue collaboration that involved establishment of an interdisciplinary institute in a major UK university. The institute links six university departments in focusing on fundamental problems related to multiphase flow. This topic was chosen as a subject that is relevant to the company’s industry but also of sufficient coherence to form the basis of a viable body of fundamental scientific and technical work. The institute was established in 2000 under an endowment from the company. The endowment paid for a building and continues to pay for faculty (approximately one in each participating department) and support staff. The sponsored faculty participate in company internal meetings. Also the institute hosts university seminars, which bring together university researchers and company employees. “Thus, the institute acts as a tap root into what is going on throughout the university.” The institute then develops ideas for projects, ranging from risk management to fluid engineering, that are of interest to university researchers and the company. The company benefits from the projects through fresh awareness of the problems it faces and alternative scientific approaches. This awareness is incorporated into individual in-house projects, but it is hard to track the impact of individual university projects. “The best evidence is to have many anecdotes as to the utility of engaging with the institute and the number of groups who wish to work with the institute. … [And] the use has been growing.” Another goal of the institute has been to raise the reputation of the oil and gas industry among faculty and students.

“[However,] issues arise around IP which can be difficult and take a long time to resolve. Universities generally are under too much pressure to look for money from IP. They have the view that sponsored research will, by definition, have value in the outcomes. They have no awareness of what it takes to create value from IP. The technical discussions are easy by comparison, but IP has becoming almost too difficult in every case -- in all universities. The IP barriers are off-putting at the outset, and company staff are de-motivated to spend time on them, because they will get no credit at their internal corporate reviews for dealing with them. Paying FEC is an issue – despite the fact that the endowment has covered the building. This can impact negatively at the budgetary level.”

The respondent noted that other major collaborations are more tightly constrained in terms of scope and objectives, and that, for the foreseeable future, the company cannot undertake more than two such initiatives.
