# Supplementary material for: Industry-University Collaborations in Canada, Japan, the UK and USA – With Emphasis on Publication Freedom and Managing the Intellectual Property Lock-Up Problem
Source: PLoS One. 2014 Mar 14;9(3):e90302. doi: 10.1371/journal.pone.0090302 (PMC3954545; doi:10.1371/journal.pone.0090302)
Supplement: Note S1 — 4-country comparison of university license vs industry sponsored research revenue. (DOCX) [file pone.0090302.s021.docx]

Note S1:

The following table compares industry sponsored research funding with license revenue. US, Canadian and Japanese license revenue includes income from cashed in equity. Japanese and UK values are converted to US dollars at Dec. 2013 exchange rates. Canadian values are in Canadian dollars.

|  | License revenue | Industry sponsored research | Year | Data source* |
| --- | --- | --- | --- | --- |
| Canada | $59M | $795M | 2010 | AUTM [13] |
| Japan | $17M | $450M | 2011 | MEXT [14] |
| UK | $122M | $1,029M | 2010-11 | HEFCE [15] |
| US | $2,600M | $4,100M | 2012 | AUTM [16] |

* See references in main text, to which reference numbers refer.
