# Supplementary material for: Industry-University Collaborations in Canada, Japan, the UK and USA – With Emphasis on Publication Freedom and Managing the Intellectual Property Lock-Up Problem
Source: PLoS One. 2014 Mar 14;9(3):e90302. doi: 10.1371/journal.pone.0090302 (PMC3954545; doi:10.1371/journal.pone.0090302)
Supplement: Note S2 — Examples of how de novo research collaborations are initiated and how past personal connections are not always supportive of innovative collaborations. (DOCX) [file pone.0090302.s022.docx]

Note S2:

One successful UK spin-off was founded only after the founder tried unsuccessfully to persuade former colleagues in a large computer company about the merits of his invention. It went on to develop cross-platform virtualization software that allows applications created for one CPU and OS to run on various platforms without modifications to the original source codes or binaries.

Directors for external programs played key roles in initiating Nortel’s collaborations with several North American universities and also Johnson and Johnson’s Focused Funding and Seed Fund Programs. Self-introductions at academic conferences were a catalyst for many of the Japanese collaborations. Occasionally, trade associations, programmer networks, and public agencies helped match universities with company needs. The UK’s Knowledge Transfer Partnerships (KTP) program brings together companies and universities that are unfamiliar with collaborating.
