# Supplementary material for: Industry-University Collaborations in Canada, Japan, the UK and USA – With Emphasis on Publication Freedom and Managing the Intellectual Property Lock-Up Problem
Source: PLoS One. 2014 Mar 14;9(3):e90302. doi: 10.1371/journal.pone.0090302 (PMC3954545; doi:10.1371/journal.pone.0090302)
Supplement: Note S3 — Open Innovation Principles (Kauffman Foundation). (DOCX) [file pone.0090302.s023.docx]

Note S3:

These Principles are available at <http://sites.kauffman.org/pdf/open_collaboration_principles_12_05.pdf> (accessed 22 Dec. 2013). See also the following 19 Dec. 2005 news release: Twelve Leaders Adopt Principles to Accelerate Innovation, at <http://www-03.ibm.com/press/us/en/pressrelease/19061.wss> (accessed 22 Dec. 2013).
