# Supplementary material for: Industry-University Collaborations in Canada, Japan, the UK and USA – With Emphasis on Publication Freedom and Managing the Intellectual Property Lock-Up Problem
Source: PLoS One. 2014 Mar 14;9(3):e90302. doi: 10.1371/journal.pone.0090302 (PMC3954545; doi:10.1371/journal.pone.0090302)
Supplement: Note S4 — Reasons for excluding five organizations from the analysis, even though they engaged in relationships with universities akin to research collaborations. (DOCX) [file pone.0090302.s024.docx]

Note S4:

These five excluded companies/initiatives are:

- Forest Products (FP) Innovations, a private non-profit Canadian industry research association, that facilitated a collaboration between a startup featured in the analysis and a Canadian university.
- Aetos Technologies, a technology development company that holds rights to a portfolio of discoveries from an American university. The interview with Aetos discussed three spin-offs from the university that are included in the analysis and that were financed in part by Aetos and that Aetos was instrumental in forming.
- the three companies/initiatives referenced by the last two bullets under “atypical” collaborations: the public broadcaster, the risk network initiative, and one of this initiative’s main corporate backers. These interactions did not raise issues germane to the subsequent analysis.
