# Supplementary material for: Industry-University Collaborations in Canada, Japan, the UK and USA – With Emphasis on Publication Freedom and Managing the Intellectual Property Lock-Up Problem
Source: PLoS One. 2014 Mar 14;9(3):e90302. doi: 10.1371/journal.pone.0090302 (PMC3954545; doi:10.1371/journal.pone.0090302)
Supplement: Note S5 — More on “engineering – ICT” categorization. (DOCX) [file pone.0090302.s025.docx]

Note S5:

Primarily hardware related projects, particularly those related to semiconductor chip design, etc., were not included in this subcategory. Rather they were classified as general engineering projects. However this subcategory does include telecommunications projects with a strong software component. In this case, attribution was assigned solely to Engineering – ICT rather than split between Engineering – ICT and software.
