# Supplementary material for: Industry-University Collaborations in Canada, Japan, the UK and USA – With Emphasis on Publication Freedom and Managing the Intellectual Property Lock-Up Problem
Source: PLoS One. 2014 Mar 14;9(3):e90302. doi: 10.1371/journal.pone.0090302 (PMC3954545; doi:10.1371/journal.pone.0090302)
Supplement: Note S6 — More on “engineering – gov’t” categorization. (DOCX) [file pone.0090302.s026.docx]

Note S6

If a project had clear civilian as well as potential defense related applications, for example those involving BAE and Rolls Royce, they were not placed in this subcategory. In the case of one US startup pursuing both defense and civilian applications, the project was allocated 50% to this category.
