# Supplementary material for: Industry-University Collaborations in Canada, Japan, the UK and USA – With Emphasis on Publication Freedom and Managing the Intellectual Property Lock-Up Problem
Source: PLoS One. 2014 Mar 14;9(3):e90302. doi: 10.1371/journal.pone.0090302 (PMC3954545; doi:10.1371/journal.pone.0090302)
Supplement: Note S7 — More on split classifications between technical fields. (DOCX) [file pone.0090302.s027.docx]

Note S7

For example, in the case of a collaboration to develop new semiconductor materials, the company would be deemed to be participating 50%, each, in an engineering and chemistry-materials collaboration. While in the case of a biomedical device collaboration, the company’s activity would be allocated 50% to biology and 50% to engineering. Many of the collaborations involved some software development on the part of the university researchers. However, in the case of an engineering, chemical or biomedical company, unless the respondent specifically said that the collaboration primarily involved software development, the attribution would be entirely to engineering, chemistry or biology – and if the collaboration was primarily software orientated, then it would be attributed half to software and half to engineering, chemistry or biology. In other words, the green portion of Fig. 2 vastly underestimates the proportion of software-related R&D in these collaborations. (See also Note S5.)
