# Supplementary material for: Industry-University Collaborations in Canada, Japan, the UK and USA – With Emphasis on Publication Freedom and Managing the Intellectual Property Lock-Up Problem
Source: PLoS One. 2014 Mar 14;9(3):e90302. doi: 10.1371/journal.pone.0090302 (PMC3954545; doi:10.1371/journal.pone.0090302)
Supplement: Note S8 — Contact time with university collaborators, startups compared with large companies. (DOCX) [file pone.0090302.s028.docx]

Note S8

Startup researchers (those based outside the university, not in a virtual company inside the university) tend to go to the collaborating university laboratories multiple times per week, compared with large company researchers, one or two of whom visit the university laboratories on average once every one or two months. However, two of the large Japanese companies in this survey currently station (or have recently stationed) several researchers long term in university laboratories in order to interact closely with university counterparts. This represents a high commitment of corporate resources to the collaborations. The authors know of other large Japanese companies that also station their researchers in satellite laboratories within universities.
