# Supplementary material for: Industry-University Collaborations in Canada, Japan, the UK and USA – With Emphasis on Publication Freedom and Managing the Intellectual Property Lock-Up Problem
Source: PLoS One. 2014 Mar 14;9(3):e90302. doi: 10.1371/journal.pone.0090302 (PMC3954545; doi:10.1371/journal.pone.0090302)
Supplement: Note S9 — Additional information on the KTP program. (DOCX) [file pone.0090302.s029.docx]

Note S9

KTPs are centered around new first-degree university graduates, known as KTP Associates, who work for two years as paid interns in companies to help them modernize their operations. The Associates are in frequent contact with university mentors. Associates’ salaries are largely covered by the UK Government. Often after two years, they are hired by the companies. KTP awards require that IP arising from the project be owned by the companies. In most KTP cases, patentable inventions did not arise.

All but one of the six KTP engaged respondents described important benefits. One small manufacturer said that a KTP turned the company around and made it profitable, and a major utility declared that KTP helped it to reorganize and refocus its entire R&D operation [Cases S15]. The one exception was an engineering consulting company that said it viewed KTP primarily as a recruiting tool. Only one of the six KTP collaborations involved a startup.
