# Supplementary material for: Industry-University Collaborations in Canada, Japan, the UK and USA – With Emphasis on Publication Freedom and Managing the Intellectual Property Lock-Up Problem
Source: PLoS One. 2014 Mar 14;9(3):e90302. doi: 10.1371/journal.pone.0090302 (PMC3954545; doi:10.1371/journal.pone.0090302)
Supplement: Note S10 — Examples of Canadian universities engaged in blue-sky collaborations expecting to own resulting inventions. (DOCX) [file pone.0090302.s030.docx]

Note S10

One company expressed this desire while describing a collaboration with an American university that has a well known reputation for insisting that it retain ownership over all sponsored research inventions – implying that while the company realized it could not obtain ownership of US university inventions, it nevertheless expected to obtain ownership of sponsored inventions from Canadian universities.

The other company had managed for many years to obtain ownership of a series of energy-related inventions by direct assignment of rights from the professor to the company. But it noted that recently it has had to comply with new Canadian Government funding agency rules limiting this practice.
