# Supplementary material for: Industry-University Collaborations in Canada, Japan, the UK and USA – With Emphasis on Publication Freedom and Managing the Intellectual Property Lock-Up Problem
Source: PLoS One. 2014 Mar 14;9(3):e90302. doi: 10.1371/journal.pone.0090302 (PMC3954545; doi:10.1371/journal.pone.0090302)
Supplement: Note S11 — Praise for assays developed by a research consortium under which a company engaged in a blue-sky collaboration, even though the company suggested restructuring collaborations as consultancies in order to avoid paying FEC. (DOCX) [file pone.0090302.s031.docx]

Note S11

This company, however, did appreciate that all companies in the umbrella consortium under which its blue sky collaboration occurred could use, for free, patented pharmaceutical assay systems arising from all the projects carried out under the consortium umbrella. The consortium dealt with signaling molecules on cell surfaces and their roll in physiologic processes and diseases. Within the consortium, several life science companies collaborated with researchers in a UK university to clarify these issues and to develop assays to study these molecules. The respondent indicated that the development of these assays, and the evolution of scientific understanding that accompanied development of these assays, were one of the major benefits of participating in this consortium.
