# Supplementary material for: Industry-University Collaborations in Canada, Japan, the UK and USA – With Emphasis on Publication Freedom and Managing the Intellectual Property Lock-Up Problem
Source: PLoS One. 2014 Mar 14;9(3):e90302. doi: 10.1371/journal.pone.0090302 (PMC3954545; doi:10.1371/journal.pone.0090302)
Supplement: Note S12 — Evidence that American companies acceptance of US university IP management practices is probably not due to lack of patentable discoveries emerging from such research. (DOCX) [file pone.0090302.s032.docx]

Note S12

US companies were well represented among those reporting tangible results (three out of four), so they probably were as interested as the respondents from other countries in IP rights covering blue-sky discoveries. However, they were willing to go through the standard process of negotiating license rights to these inventions.
