# Supplementary material for: Industry-University Collaborations in Canada, Japan, the UK and USA – With Emphasis on Publication Freedom and Managing the Intellectual Property Lock-Up Problem
Source: PLoS One. 2014 Mar 14;9(3):e90302. doi: 10.1371/journal.pone.0090302 (PMC3954545; doi:10.1371/journal.pone.0090302)
Supplement: Note S13 — A major UK company (not among the interviewees for this study) indicating its acceptance of American university technology management practices. (DOCX) [file pone.0090302.s033.docx]

Note S13

For example, in 2007, colleagues of PT at the Judge School of Business (Cambridge University) recorded a pilot interview with a senior research director of a major UK telecommunications company who described a broad ongoing series of collaborations with a major American university. The respondent noted that his company had been long familiar with the IP management policies of American universities and had learned to accommodate to them. He did not suggest criticisms of these policies. Rather he noted that the collaborations with this particular university had gone very well and that, whatever the effect of the IP policies, his company had been able to obtain the benefits it had hoped for from the collaborations. He did, however, note that this university is not only one of America’s best, but that its technology management office is very experienced and has a well-deserved high reputation.
