# Supplementary material for: Industry-University Collaborations in Canada, Japan, the UK and USA – With Emphasis on Publication Freedom and Managing the Intellectual Property Lock-Up Problem
Source: PLoS One. 2014 Mar 14;9(3):e90302. doi: 10.1371/journal.pone.0090302 (PMC3954545; doi:10.1371/journal.pone.0090302)
Supplement: Note S14 — The contractual basis for sponsoring companies to censor academic publications, taking as an example, the University of Tokyo's standard collaborative research contract. (DOCX) [file pone.0090302.s034.docx]

Note S14

For example, the University of Tokyo’s model contract. Article 30 reads in part:

The Party desiring public release shall notify the other Party in writing … no later than 30 days prior to the scheduled day of the release. … If the Party who is so duly notified determines that the contents of the release are likely to conflict with any of its interests that are expected to be realized in the future, it shall … notify the Releasing Party in writing of the desired modifications of the contents of the release, …. The Releasing Party shall not, without the consent of the other Party, release any portion that the other Party has objected to; provided, however, that the other Party shall not unreasonably withhold such consent.

Article 25 permits collaborative research findings to be designated as trade secrets by the company:

If Know-How (trade secrets) results from joint research, both parties will consult and identify such Know-How in writing as soon as possible. Any Know-How so identified shall not be disclosed or leaked to a third party without prior written consent. …

As noted in section 2.3 above, companies collaborating with Korean universities probably have similar ability to restrict publication of collaborative research findings [38].
