# Supplementary material for: Industry-University Collaborations in Canada, Japan, the UK and USA – With Emphasis on Publication Freedom and Managing the Intellectual Property Lock-Up Problem
Source: PLoS One. 2014 Mar 14;9(3):e90302. doi: 10.1371/journal.pone.0090302 (PMC3954545; doi:10.1371/journal.pone.0090302)
Supplement: Note S15 — Calculation and interpretation of industry co-ownership rates of Japanese university patents. (DOCX) [file pone.0090302.s035.docx]

Note S15

Approximate industry co-ownership rates of US patents granted over a 12-month period ending in March 2009 and assigned completely or in part to universities in each of the following countries are as follows:

| Canada | 10% |
| --- | --- |
| Germany | 15% |
| Japan | 60% |
| UK | 6% |
| USA | 3% |

This is based on a random sample of 40 US patents for each of the above countries that were issued from April 2008 through March 2009 in which a university in that country was either sole assignee (owner) or a co-assignee (co-owner) along with another party.

In the case of Japan, the 40 patents constituted a 32 percent sample of all the 125 US patents issued over this 12-month period to a Japanese university. In this 32 percent sample, all the co-owning companies were large Japanese manufacturers.

These findings are reported in REF [48].

This analysis was repeated for Japan only using a random sample of 68 patents from among all 525 US patents granted between June 2011 and May 2012 and assigned in whole or in part to a Japanese university (a 13 percent sample of all such patents). One third of the patents in this sample were co-owned by companies. Of these, fewer than 10 percent were non-Japanese companies or Japanese small or medium size enterprises. These findings are reported in REF [49].

The difference in numbers of patents (125 vs. 525) is due to the increase in university-owned inventions following 2004 legislation that granted Japanese national universities independent administrative status, thus enabling them to own most faculty inventions. As noted under Discussion and [Note S20], prior to 1998 university inventors usually retained ownership of their inventions and let companies that gave donations to their laboratories apply for patents on their inventions. Between 1998 and 2004 inventors began to transfer ownership to universities on a voluntary basis. However, the actual increase in patent applications filed by Japanese universities as a result of the new 2004 ownership system was not seen until 2005. Similarly, there was a delay in the upturn of joint industry-university patent applications, because the contracts that permitted research collaborations where companies and universities could apply jointly for co-invented patents were not available for signature until 2004.

Furthermore, most Japanese university patent applications are initially filed in the Japan Patent Office (JPO). Applicants can then wait up to 2.5 years before filing an application for the same patent in the US Patent and Trademark Office (PTO). The average time to grant a US patent is between two and three years. Thus the sample of 2008-2009 US patents reflects inventions reported to the universities around 2003. This was during the transitional period between inventor and university ownership, and before joint company-university patent applications began to increase to the where they accounted for the majority of university patent applications today.

The second analysis of Japanese university patents was conducted to address the above issues. Assuming that the universities took half a year to file a JPO patent application after the faculty invention report, and then 2.5 years before filing an application in the US PTO, which then took 2 years to grant a patent, the average total lag between invention report and issuance of a US patent is about five years. Thus this second sample of patents probably reflects inventions reported around 2006-2007. By this time the system of university patenting had been implemented, and joint university-industry patent applications had risen to account for over half of all Japanese university applications to the JPO. Even more significantly, such joint applications had increased to account for 60 percent or more of all international (Patent Cooperation Treaty) applications filed by Japanese universities, according to MEXT data analyzed by Watanabe [47].

Thus the finding that only one-third of the later sample of university patents are co-owned by companies was somewhat unexpected. One possible explanation is a program by the Japan Science and Technology Agency’s (JST) to pay the overseas patent application costs for inventions owned solely by Japanese universities. This program went into full effect in 2005 and peaked in 2006 when JST funded about one thousand international patent applications on behalf of Japanese universities. Universities filing international patent applications on their own (without JST or company assistance) peaked one year later in 2007 at around 2400 applications.* The 2006 and 2007 peaks in overseas patent applications very likely are reflected in the higher number of US patents issued solely to Japanese universities between June 2011 and May 2012 compared to the earlier April 2008 through March 2009 period.

Discussions with Japanese university technology management officials indicate that overseas patent applications on solely-owned university inventions have been declining in recent years, due largely to financial constraints. Thus the later sample (May 2011 through June 2012) probably includes an usually large number of US patents solely owed by Japanese universities. Thus the one-third co-ownership rate suggested by the later sample likely underestimates the actual average co-ownership rate.

This analysis has assumed that universities and companies will apply for US patents only if they think the inventions are valuable (and not simply for preventing rivals from applying for patents on the same invention) and that the patents in these samples reflect valuable university discoveries.

* Main text REF [14] and Japan Science and Technology Agency (JST). 2012. Chizai jigyō no ayumi to kongo no tenkai [History and Future Development of JST’s Intellectual Property Operations]. Presentation on 19 July at the Intellectual Property Strategy Center of JST (in Japanese).
