# Supplementary material for: Industry-University Collaborations in Canada, Japan, the UK and USA – With Emphasis on Publication Freedom and Managing the Intellectual Property Lock-Up Problem
Source: PLoS One. 2014 Mar 14;9(3):e90302. doi: 10.1371/journal.pone.0090302 (PMC3954545; doi:10.1371/journal.pone.0090302)
Supplement: Note S16 — Costs covered (and not covered) by industry funding for sponsored research in Japanese universities, and international comparisons of industry contributions to total university R&D expenditures. (DOCX) [file pone.0090302.s036.docx]

Note S16

Corporate funding for joint research in Japan does not cover the salaries of full time faculty. Usually it does not cover tuition or stipends of graduate students. It covers only a fraction of infrastructure costs – usually it does not cover building/facility construction or depreciation, computer costs, utilities, etc. The overhead rate of 30 percent is less than the 50 percent (or greater) rate that most US universities charge, and rather than being dedicated to specific salary or infrastructure costs, overhead funds are for discretionary usage by the university laboratories. Corporate funding often does cover the costs of special equipment associated with the project, rental charges in case of dedicated corporate rooms/laboratories within the university, and the salaries of a growing number of non-tenured so-called “project” faculty (see [Cases S11] Example 2). In general Japanese companies pay much less per collaborative research project than do American companies. [50,48]

The following tables shows the percentage of total expenditures for research and development performed in universities (institutions of higher education) in 2010 that was funded by business in each of the four study countries (designated in **bold**) in addition to selected other countries.

| China | 33.2 |
| --- | --- |
| Germany | 13.9 |
| South Korea | 11.3 |
| **Canada** | **7.1** |
| **USA** | **5.2** |
| **UK** | **4.1** |
| **Japan** | **2.6** |
| France | 2.0 |

Data source for all countries except Canada: National Science Board (NSB) (2014) Science and Engineering Indicators 2014. Arlington, VA: National Science Foundation. (Appendix Table 4-14, Gross expenditures on R&D, by performing and funding sectors, for selected countries: 2010). Available: <http://www.nsf.gov/statistics/seind14/index.cfm/appendix/tables.htm#c5>. Accessed 13 Feb. 2014.

For Canada:

Data for total 2010 university R&D expenditures (11,249 m CAD) are from Statistics Canada. Available: <http://www.statcan.gc.ca/tables-tableaux/sum-som/l01/cst01/scte02a-eng.htm>. Accessed 13 Feb. 2014.

Data for 2010 industry funding for university R&D (795 M CAD, industry sponsored research only) are from AUTM, reference 13 main text. See also Note S1.
