# Supplementary material for: Industry-University Collaborations in Canada, Japan, the UK and USA – With Emphasis on Publication Freedom and Managing the Intellectual Property Lock-Up Problem
Source: PLoS One. 2014 Mar 14;9(3):e90302. doi: 10.1371/journal.pone.0090302 (PMC3954545; doi:10.1371/journal.pone.0090302)
Supplement: Note S17 — Abandonment (dedication to public) of about half of joint industry-university Japanese patent applications. (DOCX) [file pone.0090302.s037.docx]

Note S17

The lock-up problem is partially alleviated, because about half of Japanese joint university-industry patent applications are abandoned after three years and essentially dedicated to the public (MEXT data compiled by Watanabe [47]). Under Japanese patent law, patent applications are not reviewed until requested by the applicants, and applicants have up to three years to make this request. So the applications for half of Japanese university-industry collaborative inventions simply lapse after three years. By this time the patent applications have been published (unless the company decided to withdraw the application within 18 months of filing), preventing the invention from being patented by anyone else. Overseas patents are not obtained. These are considered to be “pure defensive” patent applications because they are filed mainly to preserve freedom to operate by pre-empting rivals from patenting the same discovery. This may increase the patent commons and reduce the patent thicket. However, the incentives for any company, particularly a startup, to invest substantial resources to exploit the full potential of these discoveries are diminished once the discovery is publicized and patent protection is not available.
