# Supplementary material for: Industry-University Collaborations in Canada, Japan, the UK and USA – With Emphasis on Publication Freedom and Managing the Intellectual Property Lock-Up Problem
Source: PLoS One. 2014 Mar 14;9(3):e90302. doi: 10.1371/journal.pone.0090302 (PMC3954545; doi:10.1371/journal.pone.0090302)
Supplement: Note S18 — Lambert Agreement URLs. (DOCX) [file pone.0090302.s038.docx]

Note S18

The Agreements can be accessed at <http://www.ipo.gov.uk/whyuse/research/lambert/lambert-mrc/lambert-mrc-agree.htm>.

See also associated links, particularly <http://www.ipo.gov.uk/lambert-guide-notes-mrc-sched.htm#sched1> with respect to ownership

and

<http://www.ipo.gov.uk/lambert-guide-notes-mrc-exploit.htm#negot> with respect to licensing.

Accessed 22 Dec. 2013.
