# Supplementary material for: Industry-University Collaborations in Canada, Japan, the UK and USA – With Emphasis on Publication Freedom and Managing the Intellectual Property Lock-Up Problem
Source: PLoS One. 2014 Mar 14;9(3):e90302. doi: 10.1371/journal.pone.0090302 (PMC3954545; doi:10.1371/journal.pone.0090302)
Supplement: Note S19 — URLs of various Canadian universities regarding publication rights and ownership of IP arising under industry sponsored research. (DOCX) [file pone.0090302.s039.docx]

Note S19

URLs setting forth policies of various Canadian universities regarding publication rights and ownership of IP arising under industry sponsored research – and also URLs for the Engage Grants.

All accessed 22 Dec. 2013.

University of British Columbia:

<http://www.uilo.ubc.ca/pages/industry-engagement/partnering/types/cra> and related links.

<http://www.uilo.ubc.ca/sites/research.ubc.ca/files/uploads/documents/UILO/CRA_assignment7014.pdf>

University of Toronto:

<http://www.research.utoronto.ca/forms/sponsored-research-agreement/> and the link to the model Sponsored Research and Collaboration Agreements.

McGill University:

<http://www.mcgill.ca/research/researchers/industry/types/collaborative>

University of Waterloo:

<https://uwaterloo.ca/research/waterloo-commercialization-office-watco/intellectual-property>

Queen’s University:

<http://www.queensu.ca/secretariat/policies/senateandtrustees/intellectualproperty.html#principles>

The University of Alberta and the University of Western Ontario are two examples of universities that require that the university own inventions arising under industry sponsored collaborative research, even though, in the case of other inventions, faculty inventors can elect whether to retain ownership or to assign their ownership rights to their university.

- <https://policiesonline.ualberta.ca/PoliciesProcedures/Policies/Patent-Policy.pdf>.
- <http://www.rso.ualberta.ca/Negotiating/IPOwnershipLicensing/Policies.aspx>.
- <http://www.uwo.ca/research/services/resources/policies/intellectual_property.html>

Engage Grants:

<http://www.nserc-crsng.gc.ca/Professors-Professeurs/RPP-PP/Engage-Engagement_eng.asp>, <http://www.nsercpartnerships.ca/media-media/bulletin-bulletin/archive-archive-eng.asp?id=8>
