# Supplementary material for: Industry-University Collaborations in Canada, Japan, the UK and USA – With Emphasis on Publication Freedom and Managing the Intellectual Property Lock-Up Problem
Source: PLoS One. 2014 Mar 14;9(3):e90302. doi: 10.1371/journal.pone.0090302 (PMC3954545; doi:10.1371/journal.pone.0090302)
Supplement: Note S20 — The evolution of Japan's pre-2004 university technology transfer system. (DOCX) [file pone.0090302.s040.docx]

Note S20

Prior to 2004, the source of funding that gave rise to an invention determined whether either (a) the nation would own it, or (b) the inventor could retain ownership. Inventors could retain ownership over inventions that could be attributed to corporate donations, but inventions arising under most other sources of funding would automatically belong to the nation. Such “national” inventions could be licensed only non-exclusively. In spite of the fact that the vast majority of research funding came form government research grants or contractually-based sponsored research from government entities, and the resulting inventions ought to have been government-owned, the vast majority of faculty inventions were attributed to donation funding. Thus prior to 2004, Japan had a *de facto* “professor’s privilege” system (i.e., faculty ownership of their inventions) in spite of a *de jure* system that would have mandated that most inventions be owned and managed by government funding agencies and licensed only non-exclusively.

Under the *de facto* system, faculty inventors let the companies that gave donations to their laboratories apply for patents. Usually the faculty members were named as inventors but their university affiliations were not noted in the applications, and assignment documents from inventors to companies were brief memoranda of understandings (in Japanese, oboegaki). Another common *quid pro quo* was that the companies would be positively inclined to hiring the professors’ students.

Beginning in 1998, some of the major national universities began to establish technology licensing organizations (TLOs), which undertook patent applications, licensing, and collection and distribution of royalties on behalf of faculty inventors who voluntarily assigned their inventions to the TLOs. Thus, the TLOs provided a transition between the former system of *de facto* inventor ownership and the system of university ownership that came into effect in 2004. During this transition, some of the TLOs successfully developed technology management capabilities and faculty began to see them as credible alternatives to either not bothering at all about IP rights, or simply letting the companies that gave them donations have these rights for free without any development commitments. [50,55]

REF 56 is the Ministry of Education (MEXT) document that urged national universities to require faculty to assign their inventions to the universities, once the achieved independent administrative status in 2004.
