# Supplementary material for: Industry-University Collaborations in Canada, Japan, the UK and USA – With Emphasis on Publication Freedom and Managing the Intellectual Property Lock-Up Problem
Source: PLoS One. 2014 Mar 14;9(3):e90302. doi: 10.1371/journal.pone.0090302 (PMC3954545; doi:10.1371/journal.pone.0090302)
Supplement: Note S21 — URLs for University of Minnesota and Penn State University policies permitting industry. (DOCX) [file pone.0090302.s041.docx]

Note S21

URLs setting forth new Pennsylvania State University and University of Minnesota policies regarding the rights of industry sponsors to university research discoveries:

<http://live.psu.edu/story/56887>

<http://www.research.psu.edu/osp/documents/award-documents/SRA%20CR%20-2012-06-16.pdf>

<http://www.research.umn.edu/techcomm/industry-sponsor.html#.UBil92Fo0vl>

Accessed 22 Dec. 2013.
