# Supplementary material for: Industry-University Collaborations in Canada, Japan, the UK and USA – With Emphasis on Publication Freedom and Managing the Intellectual Property Lock-Up Problem
Source: PLoS One. 2014 Mar 14;9(3):e90302. doi: 10.1371/journal.pone.0090302 (PMC3954545; doi:10.1371/journal.pone.0090302)
Supplement: Note S22 — URL describing University of New South Wales's Easy Access IP policy. (DOCX) [file pone.0090302.s042.docx]

Note S22

The University of New South Wales’s (NewSouth Innovation’s) Easy Access IP policy is described at <http://www.nsinnovations.com.au/sites/all/files/uploads/EAIP%20Guideline%20for%20Companies_Aug%202012%20V1.pdf>.

Accessed 13 Feb. 2014.
