# Supplementary material for: Industry-University Collaborations in Canada, Japan, the UK and USA – With Emphasis on Publication Freedom and Managing the Intellectual Property Lock-Up Problem
Source: PLoS One. 2014 Mar 14;9(3):e90302. doi: 10.1371/journal.pone.0090302 (PMC3954545; doi:10.1371/journal.pone.0090302)
Supplement: Note S23 — Large companies that explicitly stated that independent startups play a vital role in developing early stage discoveries to the point where they are attractive to large companies. (DOCX) [file pone.0090302.s043.docx]

Note S23

In addition to a multinational pharmaceutical company, these include the large companies featured in Case S3, Case S7 and Case 19.
