# Supplementary material for: Industry-University Collaborations in Canada, Japan, the UK and USA – With Emphasis on Publication Freedom and Managing the Intellectual Property Lock-Up Problem
Source: PLoS One. 2014 Mar 14;9(3):e90302. doi: 10.1371/journal.pone.0090302 (PMC3954545; doi:10.1371/journal.pone.0090302)
Supplement: Note 24 — Source for assertion that Japanese junior faculty and some graduate students are hesitant to engage in industry sponsored projects out of concern that they cannot publish their findings. (DOCX) [file pone.0090302.s044.docx]

Note S24

Based upon discussions over the past six years with graduate students and junior faculty by RK.
