# Supplementary material for: Industry-University Collaborations in Canada, Japan, the UK and USA – With Emphasis on Publication Freedom and Managing the Intellectual Property Lock-Up Problem
Source: PLoS One. 2014 Mar 14;9(3):e90302. doi: 10.1371/journal.pone.0090302 (PMC3954545; doi:10.1371/journal.pone.0090302)
Supplement: Table S1 — All companies and universities that were subjects of the case studies (listed alphabetically and unmatched). (DOCX) [file pone.0090302.s045.docx]

**Supporting Information File**

**Table S1. All companies and universities that were subjects of the case studies (listed alphabetically and unmatched)**

**Key**

Project did not involve collaborative research

Excluded from collaboration analysis (see Note S5)

Light blue-sky collaboration **Deep blue-sky collaboration**

† company was not a respondent (interviewee), but it has a close relationship with a respondent (e.g., a spin-off from a respondent) and featured prominently in the interview/case

**bold** => a startup in the sense that it was established in 1990 or later

^Δ^ In addition to being a new company (startup), this is a spin-off in that its formation was based upon technology from the university highlighted in the interview.

* university with which a collaboration was briefly mentioned or summarized, but this interaction was not highlighted in the interview. However, this "*" is applied only if no substantive collaboration was discussed in depth during the interview and only if the university was not highlighted in another interview.

( ) => number of interviews that highlighted this university as a partner (excludes instances of * listings). No ( ) => this university was highlighted in only one interview.

CANADA

| 3M Canada | Agriculture and Agri-Food Canada (AAFC) |
| --- | --- |
| Baader-Canpolar | Concordia U |
| **Bell Canada, Bell University Labs** | Dalhousie U (2) |
| **Bioriginal^Δ^** | Memorial U Newfoundland (MUN) (4) |
| **Cathexis (renamed IDBlue)^Δ^** | MIT |
| Dofasco | Old Dominion U (Virginia, USA) |
| **Distil Interactive (formerly i2Learning)^Δ^** | Red River (Community) College |
| FP Innovations | U British Columbia (2) |
| **Garrison Guitars^Δ^** | U Calgary |
| **Innovotech^Δ^** | U Manitoba |
| Motor Coach Industries | U Ottawa |
| Nortel | U Toronto* |
| **OncoGeneX^Δ^** | U Waterloo (2) |
| **OpTest Equipment^Δ^ †** | various |
| **Ostara^Δ^** | unidentified |
| **Pfizer** |  |
| Pratt & Whitney |  |
| Provincial Aerospace |  |
| **SemBioSys^Δ^** |  |
| unidentified provincial crown corporation utility |  |
| YM Biosciences |  |

21 companies total (18 included in collaborative research analysis)

JAPAN

| Advantest | Aichi Prefectural U |
| --- | --- |
| **Astellas** | Chubu U |
| **Aze** | Hiroshima U |
| Daido Steel | Hokkaido U (3) |
| **Digital Development Systems (DDS)** | Ishikawa U |
| Fujitsu | Kansai U |
| **GrandEx** **^Δ^** | Kyoto U (5) |
| Hitachi | Kyushu U* (2) |
| Komatsu | Mie U |
| **Mitsubishi Chemical** | Nagoya Inst of Technology |
| Mitsubishi Trading | Nagoya U (2) |
| Nissan | Nara Inst for Advanced Science & Tech (NAIST)* |
| **Nitride Semiconductor** **^Δ^** | Okayama U |
| Nomura | Osaka U |
| NTT DoCoMo | Shizuoka U |
| Olympus | Tohoku U (3) |
| **Peccell** **^Δ^** | Toin U |
| Resona Bank | Tokushima U |
| Rohm | Tokyo Inst of Technology |
| Sanyo | U California, Santa Barbara |
| Showa Shell | U Tokyo (6) |
|  | U Tsukuba* |
|  | Waseda U |
|  | Yokohama U (2) |
|  | various, but as clients only (3) |

21 companies total (18 included in collaborative research analysis)

UK

| Anglo American, Anglo Research | Australian Minerals Industry Research Association  (AMIRA) |
| --- | --- |
| Arup | Cambridge (8) |
| BAE | Cranfield U |
| BBC | Durham U* |
| Blairs (after purchase by Owen Quinn) | Glyndwr U* |
| BP | Harper Adams University College |
| Broadbent | Herriot Watt U* |
| Dove Nest | Imperial College |
| Electronic Arts | Lancaster U (2) |
| GSK | Liverpool John Moore U* |
| IBM | Loughborough U + others |
| **IncaDigital^Δ^** (since 2005, owned by DaiNippon) | Newcastle U |
| Jones Stroud Insulation  (since 2000 owned by Krempel Group, Germany) | Northumbria U* |
| Lees Newsome | Oxford |
| Lighthill Risk Network† | Queen Mary College |
| Lloyds of London | Smith Institute (a think tank)* |
| Logica | Tongji U (Shanghai) |
| MKW Engineering | U Birmingham* |
| Oracle Health Care systems | U Bolton* |
| Power Adhesives | U Capetown |
| Renishaw | U Central Lancaster |
| Rolls Royce | U College London (2) |
| Stent Foundations (subsidiary of Balfour Beatty) | U Dundee |
| **TeraView^Δ^** † | U East London |
| Toshiba | U Leeds |
| **Toumaz Ltd^Δ^** † | U Manchester (2) |
| **Transitive Technologies^Δ^**  (later **Transitive**  **Corporation**, bought by IBM 2009) | U Manchester Inst Science & Tech* |
| Unilever | U Michigan |
| Unilever Corporate Research, Mathematical and  Psychological Sciences Group | U New South Wales* |
| United Utilities | U Queensland |
| Waitrose owned by John Lewis partnership | U Sheffield* |
| Yorkshire Water | U Strathclyde |
|  | U Surrey* |
|  | Winchester U |
|  | Yokohama U* |

32 companies total (27 included in collaborative research analysis)

USA

| Aetos | Auburn U (4) |
| --- | --- |
| Applied Felts | Case Western Reserve |
| CH2M Hill | MIT (2) |
| Cisco Systems | Montana State U |
| **Computerized Assessments & Learning (CAL)^Δ^** | South Dakota U of Mines |
| **Cytoviva^Δ^ †** | Texas A&M |
| Dow Chemical | U Alabama, Huntsville* |
| **DuPont** | U Arizona |
| **Eagle Aqaculture^Δ^ †** | U California, Santa Barbara |
| **Falcon Protein Products^Δ^ †** | U Central Florida |
| **GATR Technologies** | U Kansas |
| General Mills | U Kentucky |
| Harris Corporation | U Minnesota |
| **HyPerComp Engineering** | U Tennessee* |
| **Johnson & Johnson** | U Wisconsin |
| Kellogg | Utah State U, Logan |
| Kimberly Clark | Virginia Tech |
| Mitsubishi Electric | various (4) |
| **Paragon Space Development** | unidentified (2) |
| Pittsburgh Paint & Glass |  |
| SRS Technologies |  |
| Toyota N. America |  |
| **WET Labs** |  |

23 companies total (20 included in collaborative research analysis)
